# Comparison of In Vitro Assays in Selecting Radiotracers for In Vivo P-Glycoprotein PET Imaging

**DOI:** 10.3390/ph10030076

**Published:** 2017-09-20

**Authors:** Renske M. Raaphorst, Heli Savolainen, Mariangela Cantore, Evita van de Steeg, Aren van Waarde, Nicola A. Colabufo, Philip H. Elsinga, Adriaan A. Lammertsma, Albert D. Windhorst, Gert Luurtsema

**Affiliations:** 1 Department of Radiology & Nuclear Medicine, VU University Medical Center, De Boelelaan 1085C, 1081 HV Amsterdam, The Netherlands; r.raaphorst@vumc.nl (R.M.R.); aa.lammertsma@vumc.nl (A.A.L.); ad.windhorst@vumc.nl (A.D.W.); 2 Department of Nuclear Medicine and Molecular Imaging, University of Groningen, University Medical Center Groningen, Hanzeplein 1, 9713 GZ Groningen, The Netherlands; heli.savolainen@hotmail.com (H.S.); a.van.waarde@umcg.nl (A.V.W.); p.h.elsinga@umcg.nl (P.H.E.); 3 Dipartimento di Farmacia-Scienze del Farmaco, Università Degli Studi di Bari, via Orabona 4, 70125 Bari, Italy; mariangelacantore@gmail.com (M.C.); colabufo@farmchim.uniba.it (N.A.C.); 4 Biofordrug slr, via Orabona 4, 70125 Bari, Italy; 5 Microbiology Systems and Biology Group, Netherlands Organisation for Applied Scientific Research (TNO), Utrechtseweg 48, 3704 HE Zeist, The Netherlands; evita.vandesteeg@tno.nl

**Keywords:** apical and basolateral membrane, bidirectional transport, blood-brain barrier, calcein-AM, tritium labeling

## Abstract

Positron emission tomography (PET) imaging of P-glycoprotein (P-gp) in the blood-brain barrier can be important in neurological diseases where P-gp is affected, such as Alzheimer´s disease. Radiotracers used in the imaging studies are present at very small, nanomolar, concentration, whereas in vitro assays where these tracers are characterized, are usually performed at micromolar concentration, causing often discrepant in vivo and in vitro data. We had in vivo rodent PET data of [^11^C]verapamil, (*R*)-*N*-[^18^F]fluoroethylverapamil, (*R*)-*O*-[^18^F]fluoroethyl-norverapamil, [^18^F]MC225 and [^18^F]MC224 and we included also two new molecules [^18^F]MC198 and [^18^F]KE64 in this study. To improve the predictive value of in vitro assays, we labeled all the tracers with tritium and performed bidirectional substrate transport assay in MDCKII-MDR1 cells at three different concentrations (0.01, 1 and 50 µM) and also inhibition assay with P-gp inhibitors. As a comparison, we used non-radioactive molecules in transport assay in Caco-2 cells at a concentration of 10 µM and in calcein-AM inhibition assay in MDCKII-MDR1 cells. All the P-gp substrates were transported dose-dependently. At the highest concentration (50 µM), P-gp was saturated in a similar way as after treatment with P-gp inhibitors. Best in vivo correlation was obtained with the bidirectional transport assay at a concentration of 0.01 µM. One micromolar concentration in a transport assay or calcein-AM assay alone is not sufficient for correct in vivo prediction of substrate P-gp PET ligands.

## 1. Introduction

P-glycoprotein (P-gp, ABCB1, MDR1) is an efflux transporter expressed with varying abundance in different tissues of the body, which affects the absorption, distribution, metabolism and elimination (ADME) of many drugs. P-gp is involved in liver canalicular biliary excretion, kidney apical renal secretion, intestinal luminal secretion and blood-brain barrier (BBB) efflux [1]. At the BBB, P-gp is expressed mainly in the capillary endothelial cells, where it transports its substrates out of the brain into the bloodstream. P-gp has a similar function and overlapping substrate specificity with breast cancer resistance protein (BCRP, ABCG2), another BBB efflux transporter [2]. P-gp can also work synergistically with metabolizing enzymes (e.g., cytochrome P450 (CYPs)), which are not only present in the liver, but also in cerebral endothelium, and as such can affect drug metabolism [3]. Because of its widespread influence on ADME profiles of drugs, P-gp has been identified as a prominent transporter protein that affects drug pharmacokinetics. All drug candidates for regulatory submissions need to be screened for substrate affinity and inhibitory potency of transporters, particularly P-gp [4].

Different cell lines are used to predict membrane permeability, oral absorption of drugs and interaction with P-gp [5,6]. Measurement of bidirectional transport in a transwell system using cell lines (over)expressing human P-gp is one of the most common and reliable in vitro assays to study P-gp interactions. This assay is typically performed in polarized cell lines that form monolayer and tight junctions, such as Madin-Darby canine kidney (MDCKII) and porcine renal epithelial (LLC-PK1) cells, transfected with the human *MDR1* gene, or human epithelial colorectal adenocarcinoma (Caco-2) cells, which endogenously express human P-gp [7]. In addition to P-gp, Caco-2 cells also express Bcrp [8] and multidrug resistance-associated protein 2 (Mrp2) [9]. Transport of a compound from the apical (A) to the basolateral (B) direction represents passive diffusion and transport from B to A P-gp mediated efflux, if P-gp is the only protein involved in the transport. An efflux ratio (ER), calculated as apparent permeability (*P*_app_) from B to A divided by apparent permeability from A to B, is generally used to describe the substrate potential. This ratio is the net result of passive diffusion and the action of P-gp and thus may reflect the contribution of P-gp to restrict flux across the membranes, rather than active efflux only. Feng et al. identified a cutoff value of 1.7 for ER in MDCK-MDR1 versus MDCK wild-type cells [10]. Compounds having a value higher than 1.7 were classified as potential P-gp substrates. The same authors identified an in vivo cutoff value of 4 for brain-to-plasma area under the curve (AUC) ratios between P-gp knockout and wild-type mice. More widely used is an in vitro cutoff value of 2, which has been reported in several publications [11,12]. By using an in vitro ER cutoff value of 2 and an in vivo ER of 3, 74% of the class 1 compounds of the Biopharmaceutics Drug Disposition Classification System (BDDCS) and 81% of the class 2–3 compounds were correctly classified as P-gp substrates [12]. Zhang et al. indicated that a drug is likely to be a P-gp substrate if its ER in vitro is higher than 2 and two or three known P-gp inhibitors significantly reduce the ER [1].

Another common in vitro test for determining interaction of a compound with P-gp is a calcein-AM assay. A non-fluorescent hydrophobic dye and P-gp substrate, calcein acetoxymethyl (AM) derivative, is normally kept out of the cells by P-gp. When P-gp is inhibited by a test compound, influx of calcein-AM will increase. Calcein-AM hydrolyzes into hydrophilic fluorescent calcein, which is then measured [10]. The calcein-AM assay is therefore a P-gp inhibition and not a substrate assay. The calcein-AM and transport assays are both useful across a broad range of apparent permeability values of test compounds, but Polli et al. showed that the transport assay is more prone to fail at high permeability, whereas the calcein-AM assay is more prone to fail at low permeability [11].

Positron emission tomography (PET) is a powerful method to investigate the functionality of P-gp in various organs in vivo. PET is a non-invasive imaging technique that can measure the concentration of a positron emitting radiolabeled molecule in a region of interest, for example in the brain. To date, several radiolabeled P-gp substrates have been used to image P-gp function at the human BBB [13]. Substrate behavior can be useful, e.g., in the case of Alzheimer’s disease (AD), where diminished P-gp function has been measured, resulting in increased brain accumulation of a PET tracer and neurotoxic compounds, such as amyloid-β [14]. On the other hand, overexpression of P-gp in the brain has been reported in epilepsy patients suffering from multidrug resistance [15]. In this case, anti-epileptic drugs that are substrates of P-gp are not able to reach the brain. For PET imaging of regional P-gp expression (as opposed to P-gp function), a PET tracer is required which binds tightly to P-gp, instead of being transported. Several attempts were made to radiolabel compounds that showed such inhibitory binding in vitro, but the PET tracers based on these compounds, such as [^11^C]tariquidar and [^11^C]elacridar, still showed substrate behavior in vivo [16]. In vitro assays usually are performed in the micromolar concentration range, whereas in vivo PET studies are carried out at nanomolar concentrations. A possible explanation is that the mechanism of interaction of the tested compounds with the P-gp protein is concentration dependent.

**Figure 1:**
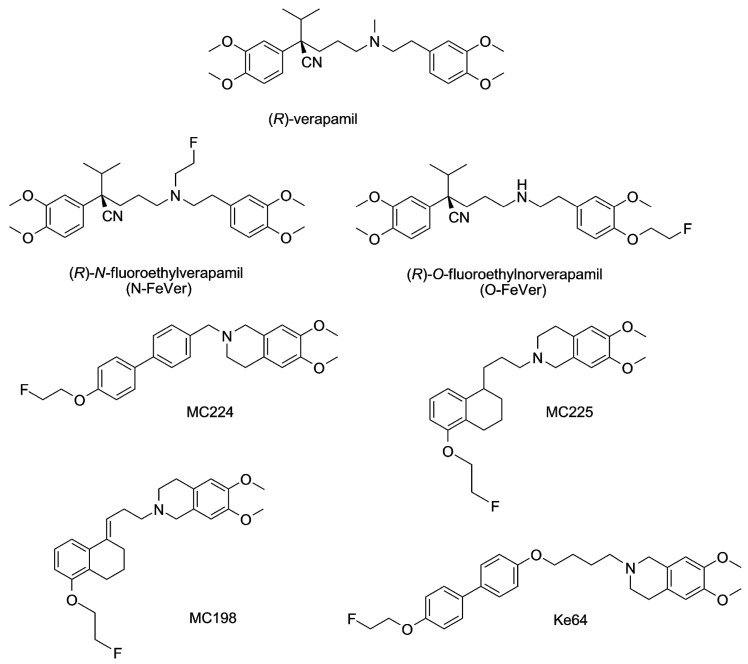
Chemical structures of all test compounds.

The purpose of the present study was to investigate the usefulness of in vitro assays for predicting the in vivo behavior of PET tracers, with a focus on the concentration dependency of ligand behavior. Previously, several fluorine-18 labeled PET ligands for in vivo measurements of P-gp function at the BBB have been developed in our labs. Fluorine-18 tracers have the advantage over carbon-11 due to a longer half-life (110 min vs. 20 min) that they can be transported to imaging facilities without an on-site cyclotron. We had existing in vivo data in mice and/or in rats for the standard substrate (*R*)-[^11^C]verapamil [17,18] and its derivatives (*R*)-*N*-[^18^F]fluoroethylverapamil (*N*-[^18^F]FeVer) and (*R*)-*O*-[^18^F]fluoroethylnorverapamil (*O*-[^18^F]FeVer) and the isoquinoline derivatives [^18^F]MC224 and [^18^F]MC225 (Figure 1) [19,20,21,22]. In this study, we included also two new fluorine-18 labeled isoquinoline derivatives, [^18^F]MC198 and [^18^F]KE64 (Figure 1), which were evaluated in vivo in mice. MC198 and KE64 are structural analogs of MC225 and MC224, respectively.

Originally, MC224 and MC225 were selected from large libraries, based on bi-directional transport data in Caco-2 cells and calcein-AM inhibition data in MDCKII-MDR1 cells, which had been acquired with non-radioactive molecules. In this study, by labeling all the molecules with tritium (^3^H, half-life 12.3 years), we could have a measurable signal even in a very low test compound concentration. Bi-directional transport assays were performed with tritiated molecules in MDCKII-MDR1 cells at three different concentrations (0.01, 1 and 50 µM). The lowest concentration (0.01 µM) best reflects the conditions seen during in vivo PET studies, whereas the higher concentrations are generally used in in vitro experiments. This set-up made it possible to evaluate whether measured ER values decreased with increasing concentration of the test compound. If so, this would indicate dose-dependent substrate transport and saturation of P-gp. In parallel experiments, cells were treated with the P-gp inhibitors ketoconazole and tariquidar (TQD) for further study of the substrate behavior of the test compounds [23,24]. The standard substrate [^3^H] digoxin was added as a control in this experiment [25]. In addition, MC224 was used as a negative control in all the transport experiments, as it did not show substrate behavior in vivo. Finally, the acquired in vitro data was compared with the in vivo data.

## 2. Results

### 2.1. Organic Synthesis

**Scheme 1:**
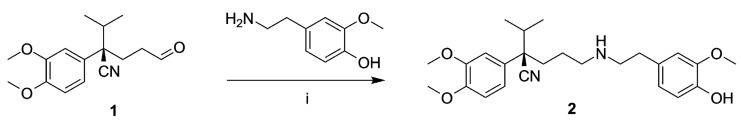
Synthesis of **2**. *Reagents*: (*i*) NaBH(OAc)_3_, Na_2_SO_4_, MeOH.

As depicted in Scheme 1, compound **2** was prepared by reductive amination using 4-(2-aminoethyl)-2-methoxyphenol and aldehyde **1**, which was synthesized in a stereoselective manner as described before [19].

**Scheme 2:**
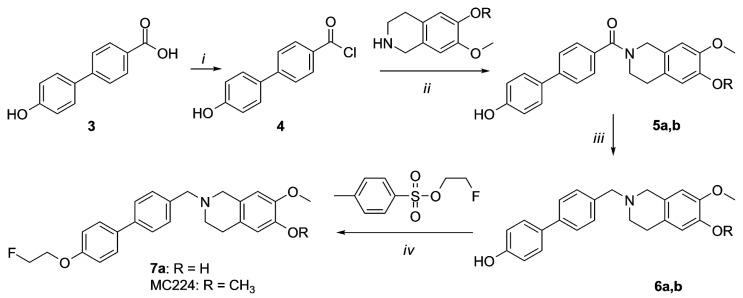
Synthesis of **7a** and MC224. *Reagents and Conditions*: (*i*) SOCl_2_, Et_3_N; (*ii*) NH_4_OH, CH_2_Cl_2_; (*iii*) LiAlH_4_, THF dry; (*iv*) NaH, DMF.

Compound **7a** and MC224 were synthesized as reported in Scheme 2. Carboxylic acid **3** was transformed into the corresponding acyl chloride **4** that was reacted with hydroxyisoquinoline to obtain amide **5a**. Final amine **7a** was obtained by reduction of amide **5a**, followed by fluoroethylation of **6a**. Synthesis of compounds **5b**–**6b** and MC224 has been reported previously and spectral data of these compounds were identical to those described previously [20].

**Scheme 3:**
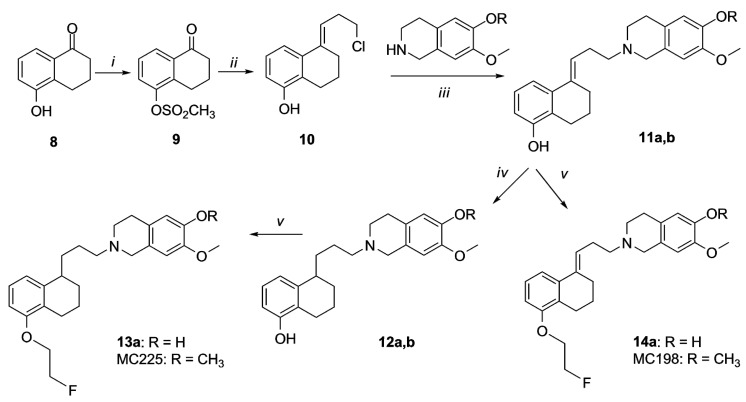
Synthesis of **13a,** MC225, **14a** and MC198. *Reagents and Conditions*: (*i*) CH_3_SO_2_Cl, Et_3_N, CH_2_Cl_2_, −10 °C; (*ii*) cyclopropylmagnesium bromide, 3 N HCl, THF; (*iii*) Na_2_CO_3_, DMF; (*iv*) H_2_, Pd/C (5%); (*v*) 2-fluoroethyl tosylate, NaH, DMF.

Compounds **13a**, MC225, **14a** and MC198 were prepared as reported in Scheme 3. Synthesis of compounds **9, 10**, **11b, 12b** and MC225 has been reported previously and spectral data of these compounds were identical to those reported [20,26]. Compounds **11a**,**b** were obtained by alkylation of chloride derivative **10** with isoquinoline. Compound **13a** was obtained by hydrogenation of **11a** and consequent fluoroethylation of compound **12a**. Compounds **14a** and MC198 were obtained by direct fluoroethylation of compounds **11a**,**b**.

**Scheme 4:**
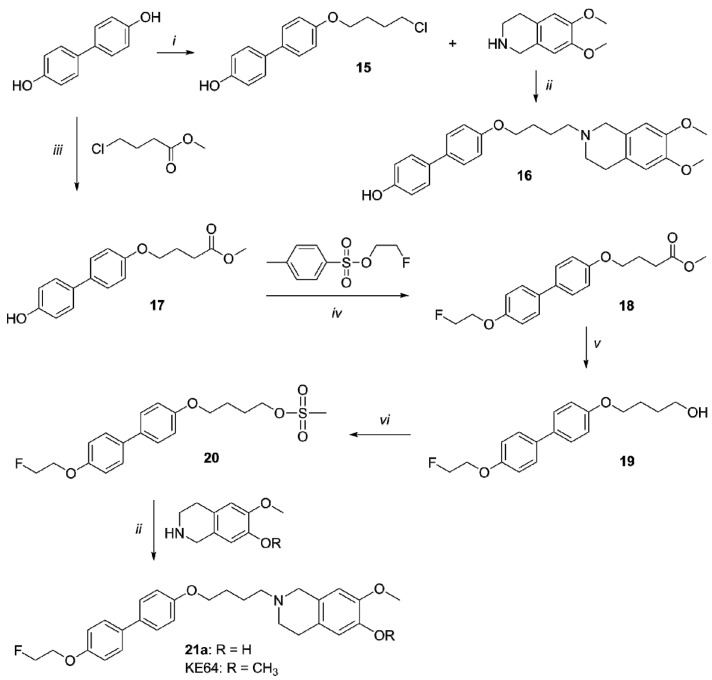
Synthesis of **16**, **21a** and KE64. *Reagents and Conditions*: (*i*) 1-bromo-4-chlorobutane, NaH, toluene; (*ii*) Na_2_CO_3_, DMF; (*iii*) NaH, THF; (*iv*) NaH, DMF; (*v*) LiAlH_4_, THF; (*vi*) MsCl, Et_3_N, CH_2_Cl_2_.

Synthesis of compounds **16**, **21a** and KE64 is depicted in Scheme 4. Dihydroxybiphenyl was reacted with methyl-4-chlorobutanoate to obtain ester **17**. Compound **18** was prepared by fluoroethylation of ester **17** and the ester function was then reducted into alcohol yielding compound **19**. Compound **19** was mesylated (compound **20**) and then condensed with 7-methoxy-1,2,3,4-tetrahydroisoquinolin-6-ol or 6,7-dimethoxy-1,2,3,4-tetrahydroisoquinoline to obtain final compounds **21a** and KE64, respectively. Synthesis of compounds **15** and **16** has been previously reported and spectral data of these compounds were identical to those reported previously [27].

### 2.2. Radiosynthesis

#### 2.2.1. Tritium Labeling

**Scheme 5:**
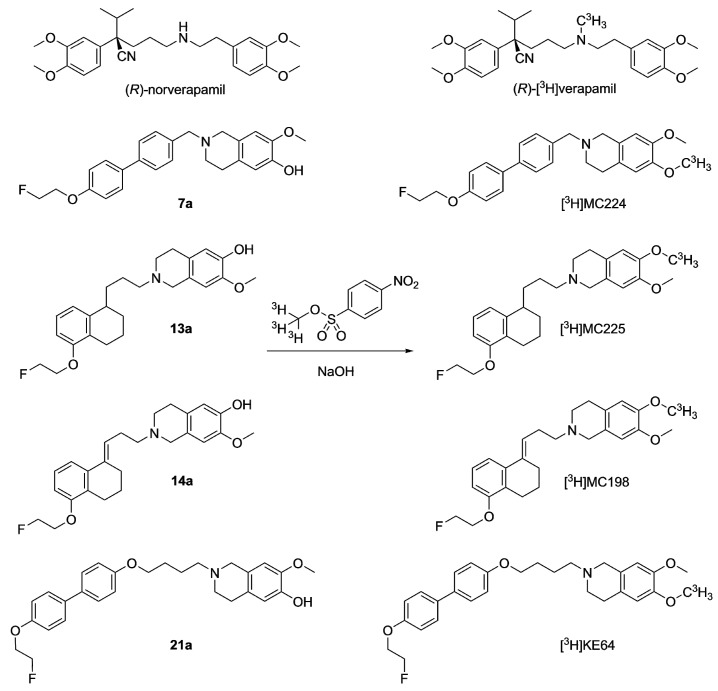
General tritium labeling of verapamil, MC224, MC225, MC198 and KE64 with [^3^H]methyl nosylate.

**Scheme 6:**
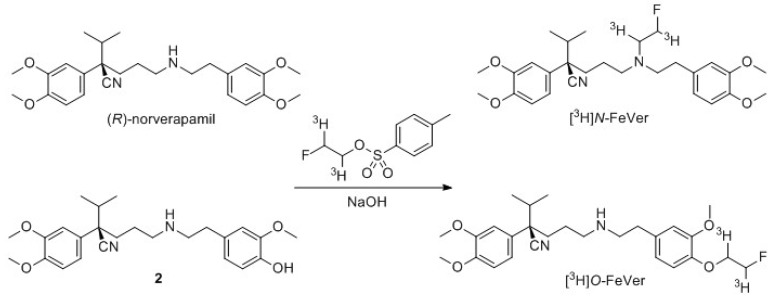
General tritium labeling of *N*-FeVer and *O*-FeVer with [^3^H]fluoroethyl tosylate.

Radiolabeling with tritium was first pursued using conditions from previous experiments with [^3^H]methyl nosylate [28]. For [^3^H]verapamil labeling, the precursor was first dissolved in MeCN with 5 M NaOH and reacted with [^3^H]methyl nosylate at 60 °C for 15 min (Scheme 5). However, milder conditions were required to reduce the formation of byproducts and therefore an overnight reaction at room temperature was used instead (25% radiochemical yield (RCY)). For the radiosynthesis of [^3^H]*N*-FeVer and [^3^H]*O*-FeVer, [^3^H]fluoroethyl tosylate was used as a labeling agent at 60 °C for 45 min, but these conditions did not yield any product. Therefore, a higher temperature of 120 °C for 45 min was used to promote the reaction towards 5.7% RCY for [^3^H]*N*-FeVer and 14% RCY for [^3^H]*O*-FeVer (Scheme 6). [^3^H]MC224 was dissolved in DMSO with 5 M NaOH and reacted with [^3^H]methyl nosylate at 60 °C for 15 min. This resulted in low yield with an unidentified byproduct. Therefore, the reaction was repeated overnight at room temperature and the yield increased under these milder conditions (26% RCY). The highest yield for [^3^H]MC225 labeling was obtained according to the standard protocol in MeCN for 15 min at 60 °C (10% RCY). For [^3^H]MC198 and [^3^H]KE64, a longer reaction time (30 min and 60 min, respectively) was needed to reach sufficient yields (3.7% and 2.3% RCY respectively). The low yield of [^3^H]MC198 was mainly due to the formation of byproducts. Most likely one of them was the isomerized form of [^3^H]MC198, as observed in the fluorine-18 labeling. The reaction mixture of [^3^H]KE64 also showed multiple unidentified byproducts. Fortunately, the two desired products could easily be purified by preparative-HPLC.

#### 2.2.2. Radiolabeling of [^18^F]MC198 and [^18^F]KE64

**Scheme 7:**
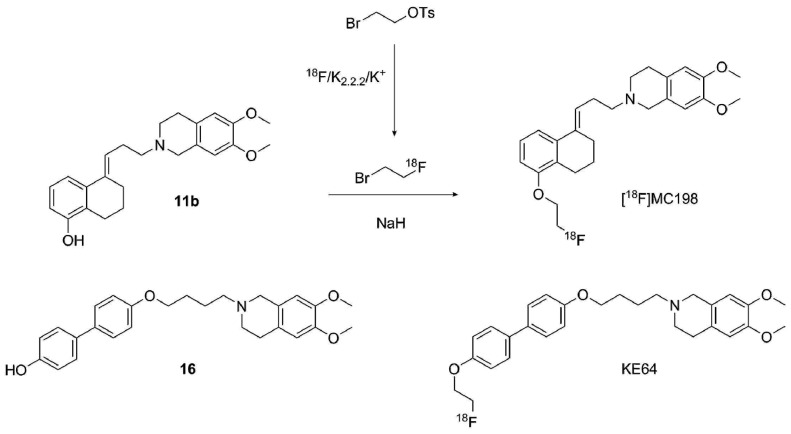
General fluorine-18 labeling of MC198 and KE64 with [^18^F]fluoroethyl bromide.

[^18^F]MC198 was challenging to label (Scheme 7), because, under basic reaction conditions, which are required for the labeling, the double bond of precursor **11b** can isomerize from the alkyl chain into the tetrahydronaphthalene ring. When the amount of NaH was increased, the amount of isomerization also increased. On the other hand, when the amount of NaH was reduced, the radiochemical yield remained low. An amount of 2 mg of NaH for 2 mg of precursor **11b** was found to yield a sufficient amount of [^18^F]MC198 (2.5% radiochemical yield, calculated from end of bombardment of ^18^F^−^) to conduct the in vivo studies.

Use of tetrabutylammonium hydroxide (TBAOH) solution (40% in water, 1 µL TBAOH and 0.5 mg of precursor) instead of NaH was also attempted, but no product was formed at all. Radiosynthesis was also attempted by starting with ethylene di(*p*-toluenesulfonate), which was first reacted with dried fluoride complex. However, the formed [^18^F]fluoroethyl tosylate was difficult to purify and using it in unpurified form could lead to side products together with the precursor and unreacted ethylene ditosylate. A Sep-Pak Silica Plus cartridge, a Sep-Pak C-18 cartridge and HPLC were used for purification, but all methods were time consuming and HPLC and silica Sep-Pak produced the intermediate in large volume. In addition, the separation in the Sep-Paks was poor. Radiosynthesis progressed more easily and faster, and produced higher yields when the distillation employing bromoethyl tosylate as precursor was used. Using 0.1 M NaOAc/MeCN 6:4 (*v*/*v*) as an HPLC eluent, the product and the isomer could be partially separated, but baseline separation was not reached with this or with other eluents tested. Identification was based on the retention time of non-radioactive MC198 in HPLC. [^18^F]MC198 was produced in 83 min with >95% radiochemical purity and >100 GBq/µmol molar radioactivity. Log D_7.4_ was measured as 2.3 according to a method reported earlier [20]. A distillation method was used without any difficulties also for [^18^F]KE64. It was generated with 6.7 ± 0.5% radiochemical yield, >97% radiochemical purity and 32 ± 17 GBq/µmol molar radioactivity in 72 min. Log D_7.4_ was determined to be 3.2.

### 2.3. In Vitro Experiments

**Figure 2:**
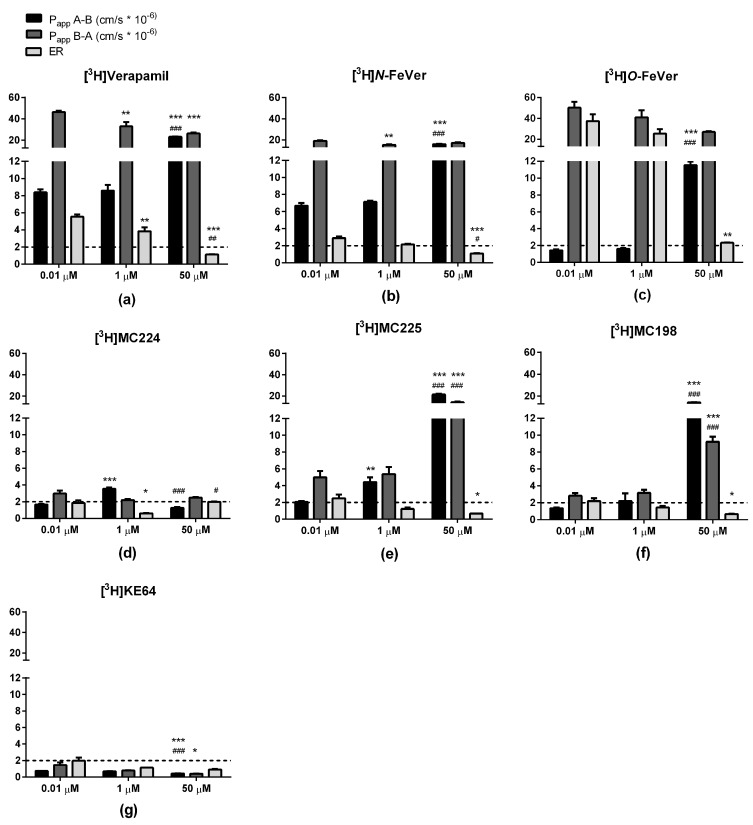
Bidirectional transport (*P*_app_ and ER) of (**a**) [^3^H]verapamil, (**b**) [^3^H]*N*-FeVer, (**c**) [^3^H]*O*-FeVer, (**d**) [^3^H]MC224, (**e**) [^3^H]MC225, (**f**) [^3^H]MC198 and (**g**) [^3^H]KE64 in MDCKII-MDR1 cells (mean ± SEM) tested at different concentrations: 0.01 µM (*n* = 6), 1 µM (*n* = 3) and 50 µM (*n* = 3). * Symbol represents a significant difference with 0.01 µM (* *p* < 0.05, ** *p* < 0.01, *** *p* < 0.001) and # symbol a significant difference with 1 µM (# *p* < 0.05, ## *p* < 0.01, ### *p* < 0.001).

**Figure 3:**
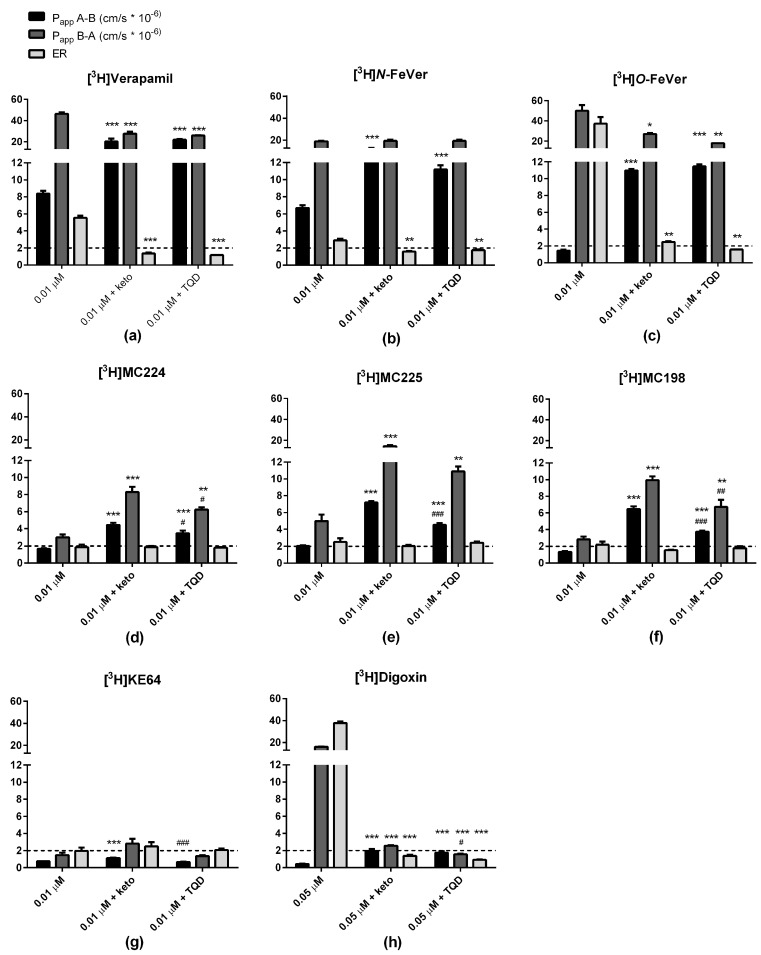
Bidirectional transport (*P*_app_ and ER) of (**a**) [^3^H]verapamil, (**b**) [^3^H]*N*-FeVer, (**c**) [^3^H]*O*-FeVer, (**d**) [^3^H]MC224, (**e**) [^3^H]MC225, (**f**) [^3^H]MC198, (**g**) [^3^H]KE64 and (**h**) [^3^H]digoxin in MDCKII-MDR1 cells (mean ± SEM) using baseline concentration of 0.01 µM (*n* = 6) with co-incubation of the P-gp inhibitor ketoconazole at 25 µM (0.01 µM + keto, *n* = 3) and tariquidar at 10 µM (0.01 µM + TQD, *n* = 3). * Symbol represents a significant difference with 0.01 µM (* *p* < 0.05, ** *p* < 0.01, *** *p* < 0.001) and # symbol a significant difference with 0.01 µM + keto (# *p* < 0.05, ## *p* < 0.01, ### *p* < 0.001).

Bidirectional transport of the tritiated test compounds ([^3^H]verapamil, [^3^H]*N*-FeVer, [^3^H]*O*-FeVer, [^3^H]MC224, [^3^H]MC225, [^3^H]MC198 and [^3^H]KE64) was investigated in MDCKII-MDR1 cells. *P*_app_ values for concentration dependent transport in both A → B and B → A direction and the ER (B → A/A → B) are shown in Figure 2. The effect of P-gp-mediated transport inhibition by ketoconazole and tariquidar is shown in Figure 3. The well-known P-gp substrate [^3^H]verapamil showed high B → A transport values (>40 × 10^−6^ cm/s) and lower A → B transport (>8 × 10^−6^ cm/s) at 0.01 µM with an ER of 5, indicating proper function of the MDCKII-MDR1 cells. With increasing concentrations of verapamil, the B → A transport and ER values decreased significantly, indicating saturation of P-gp. On the other hand, the A → B transport did not show any change from 0.01 to 1 µM, only at 50 µM the A → B transport increased significantly and the *P*_app_ values corresponded to the values observed with inhibitors ketaconazole and tariquidar. B → A transport of [^3^H]verapamil decreased significantly in combination with the inhibitors whereas A → B transport increased and consequently the ER values decreased below 2, indicating full inhibition of P-gp.

A similar behavior as with [^3^H]verapamil was observed also for [^3^H]*N*-FeVer. The only difference was that a significant change in B → A transport compared to 0.01 μM was observed only at 1 μM. [^3^H]*O*-FeVer also behaved like a P-gp substrate, with low A → B *P*_app_ values (<2 × 10^−6^ cm/s) at 0.01 µM and 1 µM, but this increased significantly at 50 µM and in the presence of inhibitors. The big difference in A → B and B → A resulted in a very high ER (>30) at 0.01 µM and 1 µM, and was still higher than 2 at 50 µM or after inhibition with ketoconazole.

[^3^H]MC224 had low *P*_app_ values in both directions (<9 × 10^−6^ cm/s) compared with [^3^H]verapamil. Different concentrations of [^3^H]MC224 did not affect the B → A transport much, but transport in both directions increased significantly due to inhibition of P-gp. The ER was <2 under all conditions, even at a tracer concentration of 0.01 µM, indicating that MC224 is not a substrate of P-gp. [^3^H]MC225 showed increased A → B and B → A values and decreased ER values at increasing concentrations and in combination with inhibitors, suggesting substrate behavior. With [^3^H]MC198, no change was observed between 0.01 and 1 µM, but A → B and B → A values increased significantly at 50 µM concentration and in combination with inhibitors. The ER of [^3^H]MC198 at 0.01 µM was slightly above 2, but below this cut-off at higher concentrations, indicating some affinity but very low capacity for P-gp mediated transport. [^3^H]KE64 did not show a clear pattern due to increasing concentrations or P-gp inhibition and in addition all *P*_app_ values were very low (<3 × 10^−6^ cm/s).

[^3^H]Digoxin transport across monolayers of MDCKII-MDR1 cells was investigated only at a concentration of 0.05 μM and in combination with inhibitors. B → A transport was high (>15) and A → B transport very low (<1) causing a really high ER (>30). Both transport values decreased significantly (to <3) due to inhibition. With both model substrates, [^3^H]verapamil and [^3^H]digoxin, A → B transport increased and B → A transport decreased due to inhibition. The difference between the two compounds was that with [^3^H]verapamil, both transport values remained high (>20). Ketoconazole and tariquidar inhibited the P-gp transport in a very similar way for all the tested compounds tested. There were only small differences within a test compound in A → B and B → A values after inhibition by either ketoconazole or tariquidar, which could be due to different P-gp binding sites of these inhibitors.

Recovery of radioactivity was determined after each experiment. [^3^H]Verapamil, [^3^H]*N*-FeVer, [^3^H]*O*-FeVer and [^3^H]digoxin showed good recovery (≥76% in all experiments, both in A → B and B → A directions). The recovery values for the remaining compounds were lower, ranging from 30 to 74% for [^3^H]MC224, 45 to 86% for [^3^H]MC225, 36 to 75% for [^3^H]MC198 and 40 to 100% for [^3^H]KE64. These low recovery values could be due to the lipophilicity of the molecules, resulting in extensive nonspecific binding to surface membrane and/or intracellular components. Log D_7.4_ values for MC224, MC225, MC198 and KE64 were 2.9, 3.0, 2.3 and 3.2, respectively, and for verapamil, *N*-FeVer and *O*-FeVer 2.6, 2.0 and 1.6, respectively [20].

**Figure 4:**
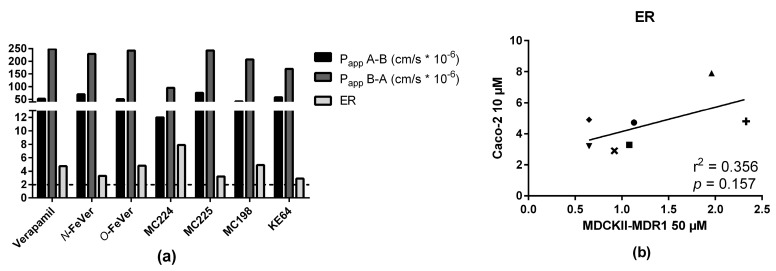
(**a**) Bidirectional transport (*P*_app_ and ER) of the test compounds in Caco-2 cells after 2 h transport using concentration of 10 µM (*n* = 1) and (**b**) correlation with ER values in MDCKII-MDR1 cells (verapamil: ●, *O*-FeVer: **+**, *N*-FeVer: ■, MC224: ▲, MC225: ▼, MC198: ♦, KE64: **×**).

In addition, the bidirectional transport of the non-radioactive molecules was evaluated in Caco-2 cells (Figure 4a). All of the compounds showed very similar results, with ER values ≥2, indicating affinity for P-gp. Remarkably, MC224 showed lower A → B and B → A values than other molecules, but still the highest ER, indicating the strongest P-gp substrate potential, although this was not observed in the in vivo data or in vitro with the tritiated molecule. Bi-directional transport of the tested compounds across monolayers of Caco-2 and MDCKII-MDR1 cells was very different. ER values obtained in Caco-2 cells did not correlate with ER of MDCKII cells at test compound concentrations of 0.01 and 1 µM. A poor correlation (*r*^2^ = 0.35) was observed between data acquired at 50 µM in MDCKII cells and at 10 µM in Caco-2 cells (Figure 4b).

A calcein-AM inhibition experiment was performed in MDCKII-MDR1 cells with non-radioactive test compounds. The lowest EC_50_ value (0.014 µM) was obtained with KE64 and the highest with *O*-FeVer (2.4 µM, Table 1), indicating that KE64 has the greatest P-gp inhibition potential.

### 2.4. In Vivo Evaluation of [^18^F]MC198 and [^18^F]KE64

**Figure 5:**
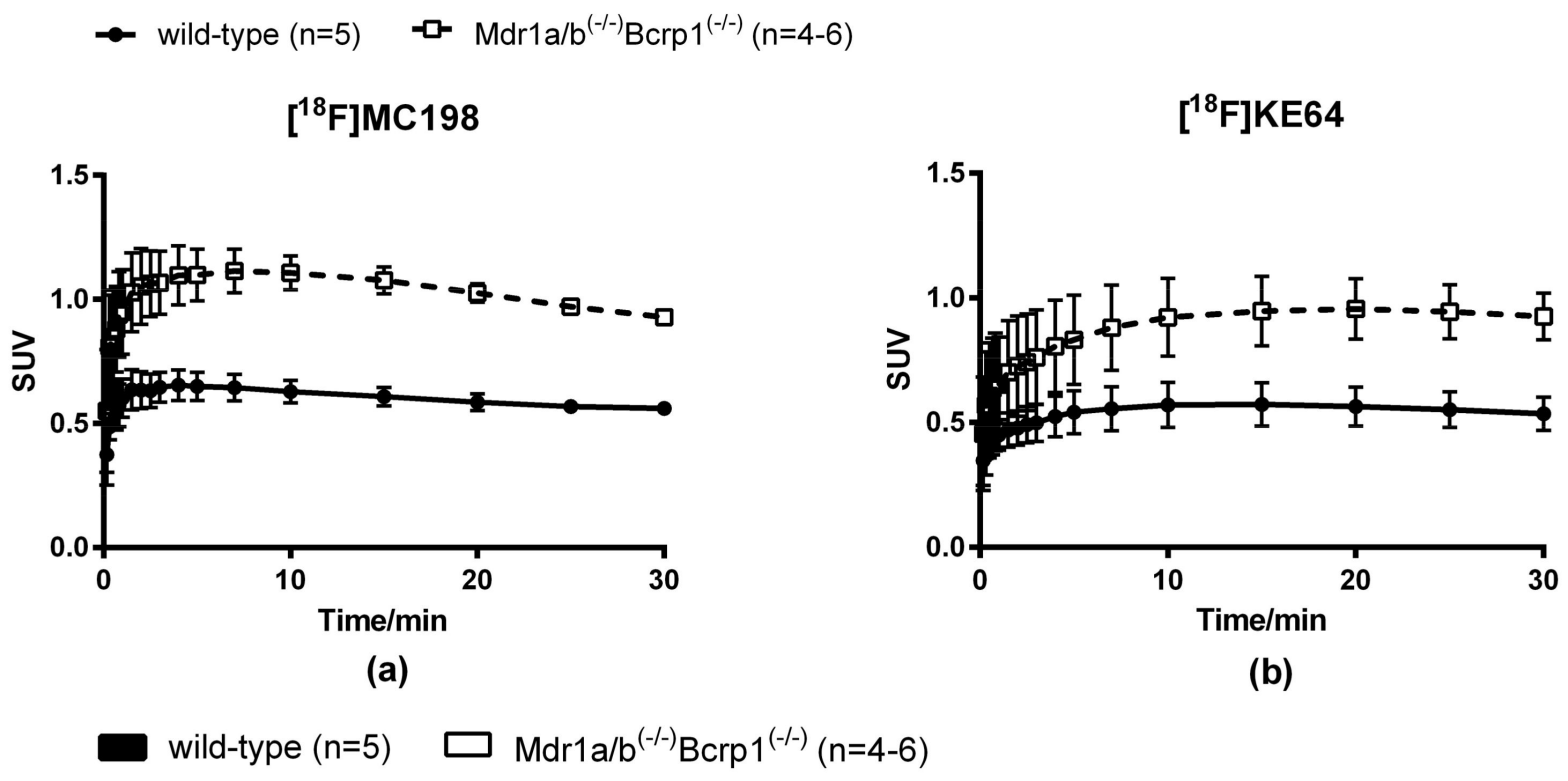

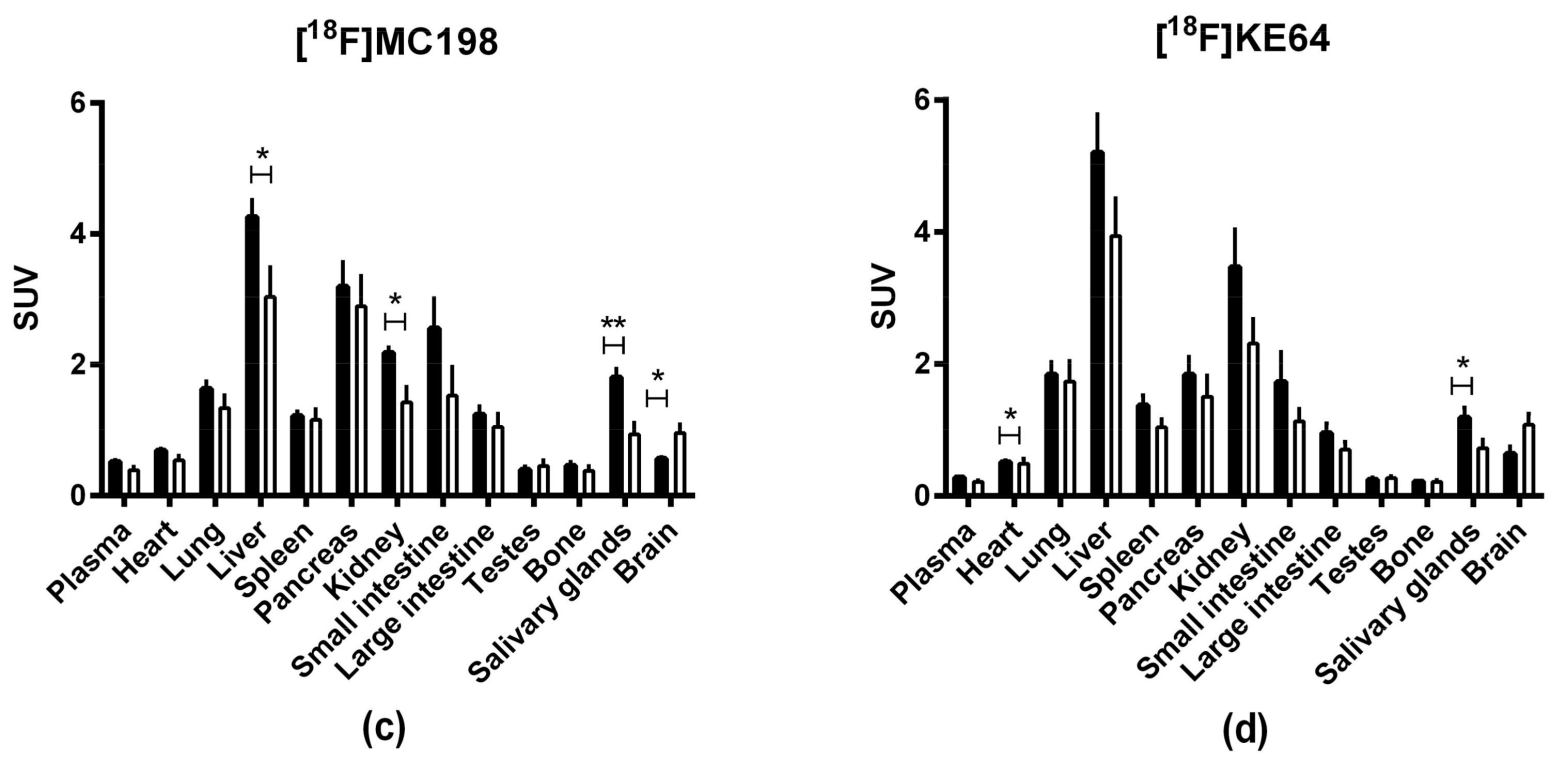
(**a**,**b**) Whole brain time-activity curves and (**c**,**d**) biodistribution profile 45 min p.i. of [^18^F]MC198 and [^18^F]KE64. Data are presented as mean ± SEM. Statistical differences are marked with a horizontal capped line (* *p* < 0.05, ** *p* < 0.01).

Radiotracer uptake in the brain was evaluated in wild-type and *Mdr1a/b*^(*−/−*)^*Bcrp1*^(*−/−*)^ knockout mice with microPET imaging. Both [^18^F]MC198 (control *n* = 5, knockout *n* = 4) and [^18^F]KE64 (control *n* = 5, knockout *n* = 6) had higher brain uptake in the knockout mice than in wild-type, indicating that they are substrates for P-gp and/or Bcrp in vivo (Figure 5a,b). The difference in whole brain time-activity curves between strains, expressed in standardized uptake value (SUV), was 1.7-fold (*P* < 0.001, comparing areas under the curve (AUC) from 0 to 30 min) for [^18^F]MC198 and 1.6-fold (*P* = 0.072) for [^18^F]KE64. The biodistribution profile of both compounds (at 45 min post-injection (p.i.)) was similar, with the highest uptake in liver, kidney, pancreas and small intestine (Figure 5c,d). Radioactive metabolites in plasma and brain at 45 min p.i. were analyzed using radio-TLC. Both compounds showed significant and rather rapid metabolism, the fraction of intact parent compound in the plasma at 45 min was only 34% and 26% for [^18^F]MC198 and [^18^F]KE64, respectively. In the brain, more metabolites were detected for [^18^F]MC198 than for [^18^F]KE64, i.e., 11 versus 5% metabolites of total radioactivity, respectively.

### 2.5. In Vitro—In Vivo Correlation

**Figure 6:**
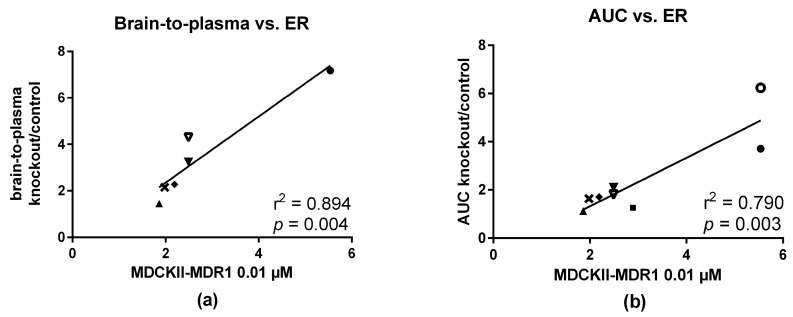
Correlation of in vitro ER values with (**a**) brain-to-plasma ratios and (**b**) AUC ratios between knockout and control mice. Brain-to-plasma ratios were calculated based on the biodistribution data 45 min p.i. using *Mdr1a/b*^(*−/−*)^*Bcrp1*^(*−/−*)^ knockout and control mice dosed with [^11^C]verapamil (●), [^18^F]MC224 (▲), [^18^F]MC198 (♦) and [^18^F]KE64 (**×**). For [^18^F]MC225 (▼), values from both *Mdr1a/b*^(*−/−*)^*Bcrp1*^(*−/−*)^ (closed symbols) and *Mdr1a/b*^(*−/−*)^(open symbols) mice were included. AUC ratios are calculated from whole brain SUV time-activity curves in the same strains as mentioned above, except that *Mdr1a/b*^(*−/−*)^ mice were used for [^18^F]*N*-FeVer (■) and these mice were used also for [^11^C]verapamil (◯) in addition to *Mdr1a/b*^(*−/−*)^*Bcrp1*^(*−/−*)^ mice (●).

In vivo evaluation was performed in control and P-gp knockout mice and for some compounds also in rats which were treated with P-gp inhibitors (Table 1) [19,20,21]. Experiments were performed in different institutions where different animal models were available and some compounds were involved in more experiments than others. The in vivo P-gp substrate potential was defined by difference in brain uptake between knockout and control animals. For radiolabeled compounds, the brain uptake can be determined either by PET imaging (time-activity curves) or by biodistribution studies (brain-to-plasma ratios). Brain-to-plasma ratios (Table 1) in knockout mice divided by brain-to-plasma ratios in control mice correlated nicely with in vitro ER values in MDCKII-MDR1 cells at both test compound concentrations of 0.01 µM (*r*^2^ = 0.89, Figure 6a) and 1 µM (*r*^2^ = 0.83, data not shown,). AUC ratios of whole brain SUV time-activity curves also correlated with ER values at these same concentrations. Correlation was slightly better at a concentration of 0.01 µM (*r*^2^ = 0.79, Figure 6b) than at 1 µM (*r*^2^ = 0.72, data not shown). [^3^H]*O*-FeVer had such high ER values at both concentrations (0.01 and 1 µM) that they were not in the same range as the others and therefore they were not included in the correlation analysis. ER of [^3^H]*O*-FeVer at a concentration of 0.01 µM was also a significant outlier according to Grubb’s test (*P* < 0.05). At a concentration of 50 µM, the correlation of ER values with both brain-to-plasma and AUC ratios was lost for all compounds. ER values obtained in Caco-2 cells (using a concentration of 10 uM) showed no correlation with the results obtained in the in vivo studies.

## 3. Discussion

This study investigated how accurately standard in vitro assays, i.e., bidirectional monolayer transport and calcein-AM assays, can predict in vivo behavior of potential P-gp substrates. For this purpose, seven different P-gp PET radiotracers were used: (*R*)-[^11^C]verapamil, [^18^F]*N*-Fever, [^18^F]*O*-Fever, [^18^F]MC224, [^18^F]MC225, [^18^F]MC198 and [^18^F]KE64. In vivo evaluation was performed in transporter knockout and control mice and/or in rats treated with the P-gp inhibitor tariquidar. Initial in vitro characterization was performed using non-radioactive compounds in combination with both the calcein-AM assay in MDCKII-MDR1 cells and the bidirectional transport assay in Caco-2 cells. The main finding was that in vivo behavior was not the same for all compounds as that observed in vitro for their non-radioactive analogs. Therefore, an attempt was made to improve the predictive value of the in vitro data by identifying more appropriate assays, focusing on the use of a bidirectional transport assay with tritium labeled compounds in MDCKII-MDR1 cells.

Due to differences between the Caco-2 and MDCK-MDR1 cell lines, the P-gp mediated transport of compounds may differ, as was observed for example with higher efflux of [^3^H]digoxin in MDCK-MDR1 cells than in Caco-2 cells [6]. The transport assay in Caco-2 cells, performed only at a relatively high test compound concentration (10 µM), did not sufficiently assess the differences between test compounds and incorrectly classified MC224 as a P-gp substrate (Figure 4).

### 3.1. Verapamil, N-FeVer and O-FeVer Behavior

Verapamil and its derivatives *N*-FeVer and *O*-FeVer showed comparable results in the transport experiments. Passive diffusion (A → B transport) increased with increasing concentrations of the compound and after treatment with inhibitors, which is an indirect consequence of the inhibition of P-gp. More compound is able to pass through the cell, without interference of P-gp, since it is not directly transported back to the apical side, resulting in a higher A → B transport. The increased A → B transport caused a decrease in the ER (Figure 2 and Figure 3). In the concentration assay, *O*-FeVer transport was affected substantially only at a concentration of 50 µM and in the Calcein-AM measurement it had the highest EC_50_ value (2.4 µM. Table 1). P-gp efflux, the B → A transport, and ER values were the highest of all the tested compounds at concentrations of 0.01 and 1 µM. This means that *O*-Fever was the strongest substrate and weakest inhibitor of P-gp in vitro. Only at 50 µM a decrease in ER was found, which implies that, for a full assessment of the concentration dependent behavior, more concentrations between 1 and 50 µM would be needed for this compound. Compared with *N*-FeVer and verapamil, low A → B transport (<2 × 10^−6^ cm/s) of *O*-FeVer at 0.01 and 1 µM indicates low passive diffusion and/or high P-gp affinity, which could be explained by the low log D_7.4_ of 1.6. This effect is also observed in vivo with slowly increasing brain uptake and absence of an initial peak of activity [19]. However, verapamil was still the strongest substrate in vivo as it had the highest brain-to-plasma and AUC ratios, both in mice and rats (Table 1). *N*-FeVer actually showed a rather low AUC ratio in mice (1.2), but this was higher in rats (3.7), indicating species differences. With *O*-FeVer, it was the other way around. Both *N*-FeVer and *O*-FeVer showed rapid in vivo metabolism. After 5 min, only 46 and 20% of the total activity in plasma represented parent *N*- and *O*-FeVer, respectively, whilst for verapamil it was 88%. However, the formed metabolites are not expected to interact with P-gp, since they are not fluorine-18 analogs of the known [^11^C]verapamil metabolite and P-gp substrate [^11^C]D617 [19,29].

### 3.2. MC224, MC225, MC198 and KE64 Behavior

Compounds MC224, MC225, MC198 and KE64 are all structural analogs. The difference between MC225 and MC198 is only a double bond in MC198 in the middle of the molecule versus a single bond in MC225 (Figure 1). The transport assay in MDCKII-MDR1 cells yielded very similar ER values for both compounds, although A → B and B → A values were a bit higher for MC225 (Figure 2e,f and Figure 3e,f). The calcein-AM assay identified MC198 as a slightly more potent P-gp inhibitor (Table 1). However, MC225 had higher knockout/control brain-to-blood and AUC ratios in mouse brain in vivo than MC198, it was metabolically more stable and showed fewer radioactive metabolites in the brain [20]. MC198 was also difficult to label due to isomerization of the double bond. Thus, MC225 had better characteristics for in vivo PET imaging than MC198, although the in vitro assays predicted them to be fairly equal.

MC224 has a shorter alkyl chain, with only one carbon atom separating the isoquinoline and biphenyl structures, than KE64 which has a 4-membered alkyl chain connected with an ether bond (Figure 1). In case of P-gp substrates, the in vitro ER values are expected to decrease after inhibition of P-gp. This was not observed for either MC224 or KE64. There was also no clear concentration dependent transport. The calcein-AM assay yielded EC_50_ values for MC224, which were in the same range as for MC225 and MC198 (Table 1). KE64, on the other hand, had the lowest EC_50_ value of all tested compounds (0.014 µM), suggesting the best inhibitory properties. MC224 was the only compound showing negative results both in vitro and in vivo. KE64, on the other hand, was the only compound out of the seven tested molecules that had conflicting in vitro and in vivo results. It was not a clear substrate in vitro, but at tracer concentrations, higher brain uptake (1.6-fold) was observed in *Mdr1a/b*^(*−/−*)^*Bcrp1*^(*−/−*)^ compared with wild-type mice, indicating that KE64 is a moderate P-gp substrate in vivo. However, the in vitro transport results of KE64 may not be very reliable due to low *P_app_* values obtained and extensive binding to cellular components and consequently low recovery values.

### 3.3. Calcein-AM Assay

A calcein-AM experiment alone would not have been sufficient to classify the molecules as P-gp substrates or inhibitors, as it can falsely indicate affinity for P-gp, especially in case of compounds with low *P*_app_ values, such as for MC224 [11]. However, if the calcein-AM assay is performed in combination with a transport experiment at different concentrations of the test compound, the calcein-AM assay can give valuable additional information. Solely based on the calcein-AM experiment, KE64 would have been classified as a P-gp inhibitor. As no in vivo inhibition experiments with increasing concentrations of KE64 or any other compounds were performed, it is difficult to predict whether they could be used as inhibitors at high dose. In vitro almost all molecules showed a decrease in ER at high concentrations, indicating saturation of transport. When the concentration is high enough, P-gp will be saturated and efflux is decreased. At least 3 different concentrations should be used to see a trend in the ER values. An advantage of the calcein-AM assay is that it can be automated, as it produces a readout (fluorescence) that is suitable for high throughput screening. The transport assay is more laborious. However, tritiated molecules are very easy to use in this assay, as their concentration can be determined by liquid scintillation counting, which increases throughput. Due to the high sensitivity of this technique, it is possible to use very low concentrations of the tritiated molecules in the assays. In addition, calculation of the recovery of radioactivity after the experiment provides an indication of the extent of nonspecific binding. On the other hand, the synthesis of precursor molecules for tritium labeling and the development of a labeling method resulting in sufficient radiochemical purity and yield of the labeled product may be time consuming. Concentrations of the non-radioactive compounds usually are measured with liquid chromatography (LC) combined with mass spectrometry (MS), which requires the development of an analytical method and measurement of a standard curve. Of course, both transport and calcein-AM assays predict only interaction with P-gp, but they do not take into account other factors important for PET tracers such as BBB penetration and metabolism.

### 3.4. In Vitro and In Vivo Correlation

The in vitro transport assay in MDCKII-MDRI cells, in combination with an ER cutoff value of 2 at a test drug concentration of 0.01 µM, predicted substrate behavior in vivo. In vitro and in vivo ER valueswere nicely correlated (Figure 6), even though the cell line is transfected with a human P-gp gene and the in vivo data are obtained in rodents. Similar results have also been reported by Adachi et al. in Caco-2 and LLC-MDR1 cells [30]. Feng et al. reported a good correlation of ER values determined in human and mouse mdr-transfected MDCK cell lines [10], but Yamazaki et al. found that results obtained in mice were better correlated with apparent permeability determined in a mouse than in a human cell line [31]. It is unclear whether these results reflect true species differences or solely the absence of mdr1b in the cells, since the mouse P-gp isoforms mdr1a and mdr1b are known to possess distinct functional characteristics [32].

Deciding on a clear cutoff value for P-gp substrates was more difficult in vivo than in vitro, as only a small number of compounds was included in the present study and differences in brain-to-plasma and AUC ratios were observed not only between different compounds, but also between animal strains and species. All compounds had higher brain-to-plasma than AUC ratios. Brain-to-plasma values were calculated only at one time point, whereas AUC represents the whole time frame of the PET scan. In this study, a cutoff value of 2 was used for both brain-to-plasma and AUC ratios to identify P-gp substrates. *N*-FeVer and MC225 had AUC ratios lower than this in *Mdr1a/b*^(*−/−*)^ mice, but since the brain-to-plasma ratios and AUC ratios in rats and in *Mdr1a/b*^(*−/−*)^*Bcrp1*^(*−/−*)^ mice were higher than 2, they can still be considered as substrates. MC198 and KE64 can be considered as weak substrates.

## 4. Materials and Methods

### 4.1. Chemicals

All reagents and solvents were obtained from commercial suppliers: Merck (Darmstadt, Germany), Sigma-Aldrich (St. Louis, MO, USA), Rathburn (Walkerburn, UK), Selleckchem, (Houston, TX, USA), B. Braun (Melsungen, Germany) and ThermoFisher (Waltham, MA, USA). (*R*)-Norverapamil was a gift from Abbott Laboratories (Chicago, IL, USA). [^3^H]Methyl nosylate (3.08 GBq/µmol) and [^3^H]digoxin (1.47 GBq/µmol) were purchased from PerkinElmer Inc. (Boston, MA, USA). [^3^H]Fluoroethyl tosylate (1.13 GBq/µmol) was purchased from Novandi Chemistry AB (Södertälje, Sweden).

### 4.2. Organic Synthesis

#### General Methods

Column chromatography was performed with 1:30 (mg crude product: g silica gel) silica gel 60 Å (63–200 μm, Merck, Darmstadt, Germany) as the stationary phase. Melting points were determined in an open capillary on a Gallenkamp electrothermal apparatus (Loughborough, UK). ^1^H-NMR spectra were recorded in CDCl_3_ at 300 MHz with a Mercury-VX (Varian, Palo Alto, CA, USA) or at 250 MHz on an Avance (Bruker Billerica, MA, USA) spectrometer. All chemical shift values are reported in ppm (δ) relative to the solvent peak (7.27 for CHCl_3_). Recording of mass spectra was carried out on an HP 6890-5973MSD gas chromatograph/mass spectrometer (Agilent, Santa Clara, CA, USA); only significant m/z peaks, with their percentage of relative intensity in parentheses, are reported. Electrospray ionization mass spectrometry (ESI-MS) analyses were performed on an Agilent 1100LC/MSDtrap systemVL. Electrospray ionisation-high resolution mass spectrometry (ESI-HRMS) was carried out using a Bruker microTOF-Q instrument in positive ion mode (capillary potential of 4500 V). Elemental analyses (C, H, N) were performed on an Euro EA 3000 analyzer (Eurovector, Milan, Italy); the analytical results were within ±0.4% of the theoretical molecular formula values.

*(R)-2-(3,4-Dimethoxyphenyl)-5-((4-Hydroxy-3-methoxyphenethyl)amino)-2-isopropylpentane-nitrile* (**2**). (*R*)-2-(3,4-Dimethoxyphenyl)-2-isopropyl-5-oxopentanenitrile (**1**, 95 mg, 0.34 mmol) was dissolved in MeOH (2 mL) and added to a suspension of 4-(2-aminoethyl)-2-methoxyphenol (110 mg, 0.55 mmol) and Na_2_SO_4_ (1.5 g, 11 mmol) in MeOH (2 mL). The reaction mixture was stirred overnight at room temperature under argon. Next, sodium triacetoxyhydroborate (120 mg, 0.55 mmol) was added and stirred for 3.5 h at room temperature. The reaction mixture was quenched with a few drops of 1 M NaOH, extracted with EtOAc and washed with H_2_O and brine. The solvent was concentrated under reduced pressure and the product was purified by flash column chromatography on a Buchi (Flawil, Switzerland) Sepacore system (comprising of a C-620 control unit, a C-660 fraction collector, 2 C601 gradient pumps and a C640 UV detector) equipped with a Buchi Sepacore prepacked flash column of 12 g Silica using a gradient (2–5% MeOH/CH_2_Cl_2_ over 30 min) to obtain the desired product **2** as a colorless oil (30 mg, 70 µmol, 20%). ESI-HRMS: calculated for C_25_H_34_N_2_O_4_: 426.2519; 427.2657 [M + H]^+^ found. ^1^H-NMR (CDCl_3_) δ: 6.63–6.94 (m, 6H, CH_AR_), 3.90 (s, 3H, OC*H_3_*), 3.86 (s, 3H, OC*H_3_*), 3.85 (s, 3H, OC*H_3_*), 2.89 (s, 2H, CH_2_CH_2_C*H_2_*NH), 2.78 (t, 2H, *J* = 6.7 Hz, CH_2_CH_2_CH_2_NHC*H_2_*CH_2_), 2.24 (m, 2H, CH_2_C*H_2_*CH_2_NH), 2.08 (m, 3H, CH_2_CH_2_CH_2_NHCH_2_C*H_2_* and C*H*(CH_3_)_2_), 1.70 (m, 2H, C*H_2_*CH_2_CH_2_NHCH_2_CH_2_), 1.18 (d, 3H, *J* = 6.6 Hz, CH(C*H_3_*)_2_), 0.78 (d, 3H, *J* = 6.6 C(C*H_3_*)_2_).

*(6-Hydroxy-7-methoxy-3,4-dihydroisoquinolin-2(1H)-yl)(4′-hydroxybiphenyl-4-yl)methanone* (**5a**). 4-(4-Hydroxyphenyl)-benzoic acid **3** (2.0 g, 14 mmol) was refluxed with SOCl_2_ (2.0 mL, 0.30 mmol) in the presence of Et_3_N (1.0 mL, 13 mmol) for 1 h. After evaporation of SOCl_2_, the resulting acyl chloride **4** was reacted with 7-methoxy-1,2,3,4-tetrahydroisoquinolin-6-ol (2.5 g, 14 mmol) in a mixture of NH_4_OH (8 M), H_2_O and CH_2_Cl_2_ (1:1:1 *v*/*v*/*v,* 45 mL), and this mixture was stirred at room temperature for 4 h. The organic layer was separated from the aqueous layer and washed with 2 M NaOH (3 × 10 mL). The organic solution was dried over Na_2_SO_4_ and evaporated under reduced pressure. The residue was purified by silica gel column chromatography (CH_2_Cl_2_/MeOH 95:5 *v*/*v*). The yield of brown solid **5a** was 3.3 g (8.8 mmol, 64%). ESI^−^/MS *m*/*z* calculated for C_23_H_21_NO_4_: 375; found 374 [M − H]^−^. ESI^−^/MS/MS *m*/*z*: 359 (97), 223 (57), 212 (98).

*2-((4′-Hydroxybiphenyl-4-yl)methyl)-7-methoxy-1,2,3,4-tetrahydroisoquinolin-6-ol* (**6a**). A suspension of LiAlH_4_ (170 mg, 4.4 mmol) in dry THF (50 mL) was stirred for 10 min. A solution of amide **5a** (550 mg, 1.5 mol) in dry THF (20 mL) was added to the suspension. The mixture was refluxed for 2 h. Water (20 mL) was added until the effervescence ceased. The aqueous layer was extracted with Et_2_O (3 × 20 mL), the organic layer was separated, dried over Na_2_SO_4_ and evaporated under reduced pressure. The residue was purified by silica gel column chromatography (CH_2_Cl_2_/MeOH 9:1 *v*/*v*). The yield of white solid **6a** was 490 mg (1.3 mmol, 92%). ESI^−^/MS *m*/*z* calculated for C_23_H_23_NO_3_: 361; found 360 [M − H]^−^. ESI^−^/MS/MS *m*/*z*: 345 (100), 168 (26).

*2-((4′-(2-Fluoroethoxy)biphenyl-4-yl)methyl)-7-methoxy-1,2,3,4-tetrahydroisoquinolin-6-ol* (**7a**). A suspension of NaH (60%, 24 mg, 1.0 mmol) in dry DMF (3 mL) was stirred at room temperature for 10 min. A solution of phenol precursor **6a** (1.0 mmol) in DMF (1 mL) was added and the solution was stirred for 1 h. A solution of 2-fluoroethyl tosylate was added (440 mg, 2.0 mmol) in DMF (1 mL) and the reaction mixture was stirred for 4 h. The synthesis of 2-fluoroethyl tosylate has been described previously [20]. Water was added until effervescence ceased. The solvent was evaporated under reduced pressure and the residue was partitioned between H_2_O (20 mL) and CHCl_3_ (20 mL). The organic phase was separated, the aqueous phase was extracted with CHCl_3_ (3 × 50 mL) and the collected organic fractions were dried over Na_2_SO_4_ and evaporated under reduced pressure. The residue was purified on silica gel column chromatography (CHCl_3_/MeOH 19:1 *v*/*v*) and recrystallized from MeOH/Et_2_O. The yield of white solid **7a** was 220 mg (0.54 mmol, 56%). ESI^+^/MS *m*/*z* calculated for C_25_H_26_FNO_3_: 407; found 408 [M + H]^+^. ESI^+^/MS/MS *m*/*z*: 229 (100), 183 (94).

*(E)-2-(3-(5-Hydroxy-3,4-dihydronaphthalen-1(2H)-ylidene)propyl)-7-methoxy-1,2,3,4-tetrahydroisoquinol-in-6-ol* (**11a**). A solution of chloride **10** (180 mg, 0.80 mmol), isoquinoline (450 mg, 1.7 mmol) and Na_2_CO_3_ (270 mg, 2.5 mmol) in DMF (10 mL) was refluxed overnight. The solvent was evaporated under pressure and the residue was dissolved in water (20 mL) and CHCl_3_ (20 mL). The organic phase was separated, the aqueous phase was extracted with CHCl_3_ (3 × 50 mL) and the collected organic fractions were dried over Na_2_SO_4_ and evaporated under reduced pressure. The residue was purified on silica gel column chromatography (CHCl_3_/MeOH 19:1 *v*/*v*) and recrystallized from MeOH/Et_2_O. The yield of white solid **11a** was 130 mg (0.33 mmol, 45%). ESI^+^/MS *m*/*z* calculated for C_23_H_27_NO_3_: 365; found 386 [M + Na]^+^, ESI^+^/MS/MS *m*/*z*: 371 (100), 198 (16).

*2-(3-(5-Hydroxy-1,2,3,4-tetrahydronaphthalen-1-yl)propyl)-7-methoxy-1,2,3,4-tetrahydroisoquinolin-6-ol* (**12a**). A solution of **11a** (140 mg, 0.36 mmol) in EtOH (50 mL) and a pinch of Pd/C 10% were stirred overnight at room temperature under hydrogen. The catalyst was filtered off and the solvent was evaporated under pressure. The residue was purified by silica gel column chromatography (CHCl_3_/MeOH 9:1 *v*/*v*). The yield of white solid **12a** was 110 mg (0.50 mmol, 85%). ESI^+^/MS *m*/*z* calculated for C_23_H_29_NO_3_: 367; found 368 [M + H]^+^. ESI^+^/MS/MS *m*/*z*: 218 (41), 187 (100), 165 (62).

*2-(3-(5-(2-Fluoroethoxy)-1,2,3,4-tetrahydronaphthalen-1-yl)propyl)-7-methoxy-1,2,3,4-tetrahydroisoquinol-in-6-ol* (**13a**). Compound **13a** was prepared in the same way as reported above for compound **7a**. The residue was purified on silica gel column chromatography (CHCl_3_/MeOH 9:1 *v*/*v*). The yield of yellow solid **13a** was 320 mg (0.77 mmol, 22%). ESI^−^/MS *m*/*z* calculated for C_25_H_32_FNO_3_: 413; found 412 [M − H]^−^. ESI^−^/MS/MS m/z: 392 (100). ^1^H-NMR (CDCl_3_) δ: 6.84–7.10 (m, 2H, aromatic tetrahydro-naphtalene), 6.58–6.63 (m, 2H, aromatic), 6.52 (s, 1H, aromatic), 5.6 (bs, 1H, O*H*), 4.83 (t, 1H, *J* = 3 Hz, OC*H*_2_CH_2_F), 4.66 (t, 1H, *J* = 3 Hz, OC*H*_2_CH_2_F), 4.24 (t, 1H, *J* = 3 Hz, OCH_2_C*H*_2_F), 4.14 (t, 1H, *J* = 3 Hz, OCH_2_C*H*_2_F), 3.83 (s, 3H, C*H_3_*), 3.55 (s, 2H, NC*H_2_* isoquinoline), 2.60–2.84 (m, 8H, NC*H*_2_CH_2_CH_2_CH, C*H*_2_CH_2_CH_2_CH tetrahydronaphtalene, NC*H_2_*C*H_2_* isoquinoline),1.63–1.85 (m, 9H, NCH_2_C*H*_2_C*H*_2_C*H*, CH_2_C*H*_2_C*H*_2_CH tetrahydronaphthalene).

*(E)-2-(3-(5-(2-Fluoroethoxy)-3,4-dihydronaphthalen-1(2H)-ylidene)propyl)-7-methoxy-1,2,3,4-tetrahydro-isoquinolin-6-ol* (**14a**). Compound **14a** was prepared in the same way as reported above for compound **7a.** The residue was purified on silica gel column chromatography (CHCl_3_/MeOH 9:1 *v*/*v*). The yield of yellow solid **14a** was 280 mg (0.71 mmol, 12%). ESI^−^/MS *m*/*z* calculated for C_25_H_30_FNO_3_: 411; found 410 [M − H]^−^. ESI^−^/MS/MS *m*/*z*: 390 (100), 364 (52). ^1^H-NMR (CDCl_3_) δ: 6.48–7.2 (m, 5H, aromatic), 6.0 (t, 1H, *J* = 6 Hz, NCH_2_CH_2_C*H=*C), 5.6 (bs, 1H, O*H*), 4.83 (t, 1H, *J* = 3 Hz, OC*H*_2_CH_2_F), 4.66 (t, 1H, *J* = 3 Hz, OC*H*_2_CH_2_F), 4.24 (t, 1H, *J* = 3 Hz, OCH_2_C*H*_2_F), 4.14 (t, 1H, *J* = 3 Hz, OCH_2_C*H*_2_F), 3.83 (s, 3H, C*H_3_*), 3.55 (s, 2H, NC*H_2_* isoquinoline), 2.60–2.84 (m, 8H, NC*H*_2_CH_2_CH=C, C*H*_2_CH_2_CH_2_C tetrahydronaphtalene, NC*H_2_*C*H_2_* isoquinoline), 1.63–1.85 (m, 6H, NCH_2_C*H*_2_CH*=*C, CH_2_C*H*_2_C*H*_2_C tetrahydronaphtalene).

*(E)-2-(3-(5-(2-Fluoroethoxy)-3,4-dihydronaphthalen-1(2H)-ylidene)propyl)-6,7-dimethoxy-1,2,3,4-tetra-hydroisoquinoline* (MC198). MC198 was prepared in the same way as reported above for compound **7a**. The residue was purified on silica gel column chromatography (CHCl_3_/MeOH 9:1 *v*/*v*). The yield of yellow oil MC198 was 230 mg (0.87 mmol, 15%). ESI^+^/MS *m*/*z* calculated for C_26_H_32_FNO_3_: 425; found 426 [M + H]^+^. ESI^+^/MS/MS *m*/*z*: 262 (33), 235 (100), 179 (65). ^1^H-NMR (CDCl_3_) δ: 6.48–7.30 (m, 5H, aromatic), 6.0 (t, 1H, *J* =6 Hz, NCH_2_CH_2_C*H=*C), 4.83 (t, 1H, *J* =3 Hz, OC*H*_2_CH_2_F), 4.66 (t, 1H, *J* =3 Hz, OC*H*_2_CH_2_F), 4.24 (t, 1H, *J* =3 Hz, OCH_2_C*H*_2_F), 4.14 (t, 1H, *J* =3 Hz, OCH_2_C*H*_2_F), 3.83 (s, 3H, C*H_3_*), 3.55 (s, 2H, NC*H_2_* isoquinoline), 2.40–2.84 (m, 8H, NC*H*_2_CH_2_CH=C, C*H*_2_CH_2_CH_2_C tetrahydronaphtalene, NC*H_2_*C*H_2_* isoquinoline), 1.73–1.80 (m, 6H, NCH_2_C*H*_2_CH*=*C, CH_2_C*H*_2_C*H*_2_C tetrahydronaphtalene).

*Methyl 4-(4′-hydroxybiphenyl-4-yloxy)butanoate* (**17**). Dihydroxybifenyl (1.5 g, 8.0 mmol) dissolved in dry DMF (5 mL) was added to a suspension of NaH (100 mg, 4.1 mmol) in dry DMF (5 mL). The mixture was stirred at room temperature for 20 min. Methyl-4-chlorobutanoate (3.5 g, 28 mmol) was added and the mixture was refluxed overnight. Water (20 mL) was added until the effervescence ceased. The aqueous layer was extracted with CH_2_Cl_2_ (3 × 20 mL), the organic layer was separated, dried over Na_2_SO_4_ and evaporated under reduced pressure. The residue was purified on silica gel column chromatography (CH_2_Cl_2_). The yield of yellow solid **17** was 450 mg (1.57 mmol, 20%). ESI^−^/MS *m*/*z* calculated for C_17_H_18_O_4_: 286; found 285 [M − H]^−^. ESI^−^/MS/MS *m*/*z*: 184 (100).

*Methyl 4-(4′-(2-Fluoroethoxy)biphenyl-4-yloxy)butanoate* (**18**). Compound **18** was prepared in the same way as reported above for compound **7a**. The residue was purified on silica gel column chromatography (CH_2_Cl_2_). The yield of white solid **18** was 230 mg (0.78 mmol, 45%). ESI^+^/MS *m*/*z* calculated for C_19_H_21_FO_4_: 332; found 355 [M + Na]^+^, ESI^+^/MS/MS *m/z*: 295 (10), 205 (18).

*4-(4′-(2-Fluoroethoxy)biphenyl-4-yloxy)-1-butanol* (**19**). Compound **19** was prepared by reduction with LiAlH_4_, following the same procedure as reported above for **6a**. The residue was purified on silica gel column chromatography (CHCl_3_/AcOEt 1:1 *v*/*v*). The yield of white solid **19** was 320 mg (0.13 mmol, 40%). ESI^+^/MS *m*/*z* calculated for C_18_H_21_FO_3_: 304; found 327 [M + Na]^+^, ESI^+^/MS/MS *m*/*z*: 245 (100). ^1^H-NMR (CDCl_3_) δ: 7.40–7.62 (m, 4H, aromatic, OCH_2_C*H*_2_F), 6.80–7.13 (m, 4H, aromatic), 5.2 (bs, 1H, OH), 4.83 (t, 1H, *J* =3 Hz, OC*H*_2_CH_2_F), 4.66 (t, 1H, *J* =3 Hz, OC*H*_2_CH_2_F), 4.24 (t, 1H, *J* =3 Hz, OCH_2_C*H*_2_F), 4.14 (t, 1H, *J* =3 Hz, OCH_2_C*H*_2_F), 3.65–3.84 (m, 4H, C*H*_2_CH_2_CH_2_C*H*_2_), 1.43–2.02 (m, 4H, CH_2_C*H*_2_C*H*_2_CH_2_).

*4-(4′-(2-Fluoroethoxy)biphenyl-4-yloxy)butyl methanesulfonate* (**20**). A mixture of alcohol **19** (390 mg, 1.5 mmol), MsCl (680 mg, 6.0 mmol) and Et_3_N (400 mg, 4.0 mmol) in CH_2_Cl_2_ (30 mL) was stirred at room temperature for 3 h. The organic phase was washed with saturated solution of NaHCO_3_ (3 × 30 mL) and 3 N HCl (3 × 30 mL). The organic phase was dried over Na_2_SO_4_ and evaporated under reduced pressure. The residue was purified on silica gel column chromatography (CHCl_3_/AcOEt 1:1 *v*/*v*). The yield of yellow solid **20** was 540 mg (1.8 mmol, 95%). ESI^+^/MS *m*/*z* calculated for C_19_H_23_FO_5_S: 382; found 405 [M + Na]^+^, ESI^+^/MS/MS *m*/*z*: 287 (100).

*2-(4-(4′-(2-Fluoroethoxy)biphenyl-4-yloxy)butyl)-7-methoxy-1,2,3,4-tetrahydroisoquinolin-6-ol* (**21a**). Compound **21a** was alkylated with 6-hydroxy-7-methoxyisoquinoline as reported above for **11a**. The residue was purified on silica gel column chromatography (CHCl_3_/MeOH 19:1 *v*/*v*). The yield of white solid **21a** was 480 mg (1.5 mmol, 68%). ESI^+^/MS *m*/*z* calculated for C_28_H_32_FNO_4_: 465; found 466 [M + H]^+^, ESI^+^/MS/MS *m*/*z*: 316 (66), 287 (87), 245 (100), 165 (99). ^1^H-NMR (CDCl_3_) δ: 7.40–7.62 (m, 4H, aromatic biphenyl), 6.80–7.13 (m, 4H, aromatic biphenyl), 6.6 (s, 1H, aromatic isoquinoline), 6.4 (s, 1H, aromatic isoquinoline), 5.2 (bs, 1H, OH), 4.83 (t, 1H, *J* =3 Hz, OC*H*_2_CH_2_F),), 4.66 (t, 1H, *J* =3 Hz, OC*H*_2_CH_2_F), 4.24 (t, 1H, *J* =3 Hz, OCH_2_C*H*_2_F), 4.14 (t, 1H, *J* =3 Hz, OCH_2_C*H*_2_F), 3.95–4.15 (m, 2 H, NC*H_2_* isoquinoline), 3.65–3.84 (m, 5H, C*H*_3_ and OC*H*_2_CH_2_CH_2_CH_2_N), 2.72–3.20 (m, 6 H, OCH_2_CH_2_CH_2_C*H*_2_N and NC*H_2_*C*H_2_*), 1.63–2.02 (m, 4H, OCH_2_CH_2_C*H*_2_C*H*_2_N).

*2-(4-(4′-(2-Fluoroethoxy)biphenyl-4-yloxy)butyl)-6,7-dimethoxy-1,2,3,4-tetrahydroisoquinoline* (KE64). KE64 was prepared by alkylation with 6-hydroxy-7-methoxyisoquinoline as reported above for **11a**. The residue was purified on silica gel column chromatography (CHCl_3_/MeOH 19:1 *v*/*v*). The yield of yellow solid KE64 was 79 mg (0.27 mmol, 11%). ESI^+^/MS *m*/*z* calculated for C_29_H_34_FNO_4_: 479; found 502 [M + Na]^+^, ESI^+^/MS/MS *m/z*: 248 (100), 179 (16).^1^H-NMR (CDCl_3_) δ: 7.40–7.62 (m, 4H, aromatic biphenyl), 6.80–7.13 (m, 4H, aromatic biphenyl), 6.4 (s, 1H, aromatic isoquinoline), 6.6 (s, 1H, aromatic isoquinoline), 4.83 (t, 1H, *J* =3 Hz, OC*H*_2_CH_2_F), 4.66 (t, 1H, *J* =3 Hz, OC*H*_2_CH_2_F), 4.24 (t, 1H, *J* =3 Hz, OCH_2_C*H*_2_F), 4.14 (t, 1H, *J* =3 Hz, OCH_2_C*H*_2_F), 4.2–4.3 (m, 2H, NC*H_2_* isoquinoline), 3.84 (s, 3H, C*H_3_*), 3.82 (s, 3H, C*H_3_*), 3.63–3.59 (m, 2H OC*H*_2_CH_2_CH_2_CH_2_N), 2.42–2.80 (m, 6H, OCH_2_CH_2_CH_2_C*H*_2_N and NC*H_2_*C*H_2_*), 1.63–2.02 (m, 4H, OCH_2_C*H*_2_C*H*_2_CH_2_N).

### 4.3. Radiochemistry

#### 4.3.1. General Methods in Tritium Labeling

[^3^H]Methyl nosylate stock solution (100–300 µL, 717 MBq/mL, 10:2 hexane/EtOAc) or [^3^H]fluoroethyl tosylate stock solution (300 µL, 139 MBq/mL, 6:4 heptane/EtOAc) was dried at 40 °C under a flow of argon. Precursor (*R*)-norverapamil, **2**, **7a**, **13a**, **14a** or **21a** was dissolved in MeCN (0.2 mL) and 5 M NaOH (5 µL) was added. This precursor solution was added to the dried [^3^H]methyl nosylate or [^3^H]fluoroethyl tosylate. Reaction temperatures and times varied for each compound. The reaction mixture was cooled down and diluted with 0.6 mL of MeOH/H_2_O (70:30) and purified by preparative HPLC. HPLC analysis was performed with an Agilent 1200 Series auto injector (Agilent Technologies, Waldbronn, Germany) and a single Jasco PU-2080Plus pump (Jasco Ltd., Great Dunmow, UK). A Reprosphere 100 C18-DE column (50 × 8 mm, 5µm) from Dr. Maisch GmbH (Ammerbuch, Germany) was used for preparative HPLC with MeOH/H_2_O/DIPA 70:30:0.1 as an eluent at a flowrate of 3 mL/min. The column eluate was monitored continuously using a Jasco UV-2075Plus absorbance UV/Visible spectrophotometer set at 254 nm and a β-Ram radiochemical detector (LabLogic, Sheffield, UK). Fractions of the eluate (3 mL) were collected and analyzed for radioactivity by liquid scintillation counting (LSC) (LKB/Wallac 1219 Rackbeta, Mount Waverley, Australia) using 5 mL of Optiphase ‘Highsafe 3’ scintillation liquid (PerkinElmer) for a 3 µL sample of an HPLC fraction. Collected fractions were diluted with 50 mL of sterile water and passed over a Waters tC18 Sep-Pak cartridge (Waters, Etten-Leur, The Netherlands), preconditioned with 5 mL of EtOH followed by 10 mL of water. The cartridge was washed with 20 mL of water, followed by elution of the product with 1.5 mL of 96% ethanol. All products were analysed for purity by analytical HPLC using a Platinum C18 column (250 × 4.6 mm, 100 Å, 5 µm) from Grace (Columbia, MD, USA) with a flow rate of 1 mL/min (MeCN/H_2_O/TFA 40:60:0.1) and the identity was confirmed by co-elution with a reference compound.

#### 4.3.2. [^3^H]Verapamil (Scheme 5)

[^3^H]Methyl nosylate (250 µL) was reacted overnight at room temperature with the precursor (*R*)-norverapamil (0.60 mg, 1.4 µmol) according to the general method. The product was purified with preparative HPLC (MeOH/H_2_O/DIPA 60:40:0.1 *v*/*v*/*v*) and [^3^H]verapamil (41 MBq) was obtained with 25% radiochemical yield (RCY) and >99% radiochemical purity.

#### 4.3.3. [^3^H]N-FeVer (Scheme 6)

Precursor (*R*)-norverapamil (0.6 mg, 1.4 µmol) was dissolved in 0.2 mL of MeCN and 10 µL of 5 M NaOH was added. This solution was added to the dried [^3^H]fluoroethyl tosylate and heated at 120 °C for 45 min. The crude mixture was purified with preparative HPLC (MeOH/H_2_O/DIPA 55:45:0.1 *v*/*v*/*v*)) and [^3^H]*N*-FeVer (2.3 MBq) was obtained with 5.7% radiochemical yield and > 99% radiochemical purity, as determined on analytical HPLC (MeCN/H_2_O/TFA 50:50:0.1 *v*/*v*/*v*)).

#### 4.3.4. [^3^H]O-FeVer (Scheme 6)

Precursor **2** (0.2 mg, 0.47 µmol) was reacted with [^3^H]fluoroethyl tosylate for 45 min at 120 °C, according to the general procedure. The crude mixture was purified with HPLC (MeOH/H_2_O/DIPA 55:45:0.1 *v*/*v*/*v*)) to give the desired product [^3^H]O-FeVer (6.0 MBq) with 14% radiochemical yield and >99% radiochemical purity.

#### 4.3.5. [^3^H]MC224 (Scheme 5)

[^3^H]MC224 was prepared using the general method, with the exception that precursor **7a** (0.5 mg, 1.2 µmol) was dissolved in DMSO (0.2 mL instead of MeCN. Compound **7a** was reacted with [^3^H]methyl nosylate (100 µL) at room temperature overnight. Product [^3^H]MC224 (18 MBq) was obtained with 26% radiochemical yield and > 99% radiochemical purity, as determined on analytical HPLC (MeCN/H_2_O/TFA 50:50:0.1 *v*/*v*/*v*)).

#### 4.3.6. [^3^H]MC225 (Scheme 5)

Precursor **13a** (0.35 mg, 0.85 µmol) was reacted with [^3^H]methyl nosylate (100 µL) for 15 min at 60 °C, according to the general method. [^3^H]MC225 (7.0 MBq) was obtained with 10% radiochemical yield and > 99% radiochemical purity, as determined on analytical HPLC (MeCN/H_2_O/TFA 50:50:0.1 *v*/*v*/*v*)).

#### 4.3.7. [^3^H]MC198 (Scheme 5)

[^3^H]MC198 was prepared according to the general method, by reacting precursor **14a** (0.2 mg, 0.49 µmol) with [^3^H]methyl nosylate (200 µL) for 30 min at 60 °C. The product (5.0 MBq) was synthesized with 3.7% radiochemical yield and high radiochemical purity (>99%).

#### 4.3.8. [^3^H]KE64 (Scheme 5)

[^3^H]methyl nosylate (150 µL) was reacted with the precursor **21a** (0.2 mg, 0.43 µmol) for 60 min at 60 °C according to the general method to give the desired product [^3^H]KE64 (2.3 MBq) with 2.3% radiochemical yield and >99% radiochemical purity, as determined on analytical HPLC (MeCN/H_2_O/TFA 50:50:0.1 *v*/*v*/*v*)).

#### 4.3.9. Radiosynthesis of [^18^F]MC198 and [^18^F]KE64 (Scheme 7)

Production and work-up of fluoride-18 was performed as described previously [20]. Radiolabeling was performed by a two-step distillation method as reported earlier using [^18^F]fluoroethyl bromide as an intermediate. Briefly, 2-bromoethyl tosylate (15 µL, 21 mg, 75 μmol) in 1,2-dichlorobenzene (1.0 mL) was added to the dried fluoride complex. Formed [^18^F]fluoroethyl bromide was distilled directly after addition of 2-bromoethyl tosylate at 90 °C during 15 min with a helium gas flow (40 mL/min) to the second vial at room temperature. For [^18^F]MC198, [^18^F]fluoroethyl bromide was reacted with precursor **11b** (2 mg, 5.3 µmol) and NaH (60% dispersion in mineral oil, 2 mg, 50 µmol) in DMF (0.5 mL) at 80 °C for 5 min. [^18^F]KE64 was prepared in the same way using **16** (1.5 mg, 3.5 µmol) and NaH (3.0 mg, 75 µmol) in DMF (0.5 mL) in a 10 min reaction at 80 °C. After completion of the reactions, HPLC eluent (0.1 M NaOAc/MeCN 6:4 (*v*/*v*), 0.5 mL) for [^18^F]MC198 or 1:1 for [^18^F]KE64) was added. The mixture was filtered through an Acrodisc syringe filter (0.4 µm, Pall, Port Washington, NY, USA) and subjected to HPLC purification utilizing a Symmetryshield RPS 5 µm 7.8 × 300 mm column (Waters, Milford, MA, USA) at a flow of 3 mL/min (UV detection at 254 nm, R_t_ of [^18^F]MC198 = 17 min and [^18^F]KE64 = 9 min). The product was collected into a bottle containing 80 mL of sterile water. Formulation was performed as described previously [20].

### 4.4. In Vitro Experiments with Tritiated Molecules

#### 4.4.1. Cell Culture

Madin-Darby canine kidney (MDCK) II cells expressing cDNAs encoding human MDR1 (MDCKII-MDR1 cells) were obtained by TNO from the Netherlands Cancer Institute. Transfected MDCKII cells were seeded on semi-permeable (pore size 0.4 µm) filter inserts (12-well Transwell plates 3401, Costar Corp, Cambridge, MA, USA) at approximately 4 × 10^5^ cells per filter insert (growth area of 1.13 cm^2^) and cultured according to the standard procedure for this cell line [33]. Cells were counted using an automatic cell counter (Millipore, Bedford, MA, USA). Cells on the inserts were cultured for three days in a total volume of 2.3 mL culture medium per well (0.5 mL apical and 1.8 mL basolateral) at approximately 37 °C in a humidified incubator containing approximately 95% air/5% CO_2_. The medium was refreshed after 48 h. It has been shown, that under these conditions the human transporter protein MDR1 localizes on the apical plasma membrane of the cells [34,35].

#### 4.4.2. Assessment of Monolayer Integrity

After three days of cell culture on Transwell, the MDCKII-MDR1 monolayers are expected to have developed a transepithelial electrical resistance (TEER) of approximately 70 Ωcm^2^ higher than background (empty filter without cells) [36]. At the start of each study, TEER was measured to assess the integrity of the monolayers of MDCKII-MDR1 cells in culture medium using the Millicell-ERS epithelial voltohmmeter (Millipore). Monolayers with a TEER value less than 55 Ωcm^2^ above background (empty filter) were excluded from the transport assay. TEER values ranged from 205 to 294 Ωcm^2^, with a mean of 253 Ωcm^2^, including the background of 125 Ωcm^2^. Cell monolayers were also inspected with a microscope before starting the experiments.

#### 4.4.3. Bidirectional Transport of Tritiated Compounds in MDCKII-MDR1 Cells

Transcellular transport studies were performed as described previously with minor modifications [37]. In brief, a stock solution of digoxin (0.1 mM) was prepared in DMSO. [^3^H]digoxin was mixed with a non-radiolabeled compound to achieve a final concentration of 0.05 µM digoxin in HBSS/HEPES in the transwells, and a radioactivity concentration of 10 kBq/mL. Stock solutions of test compounds verapamil, *N*-FeVer, *O*-FeVer, MC224, MC225, MC198 and KE64 (0.01, 1 and 100 mM) were prepared in DMSO, mixed with the tritium labeled analogs and diluted with HBSS/HEPES to final concentrations of 0.01, 1 and 50 µM, with a radioactive tracer concentration of 10 kBq/mL in the transwells.

After the integrity assessment, culture medium was removed from the filter inserts (apical compartment (A)). The inserts were washed once with warm (37 °C) HBSS, transferred to new 12-well plates which were preincubated with HBSS/HEPES for 18 h at 37 °C, to reduce non-specific binding to the plastic, and washed with warm HBSS. The MDR1-mediated apical to basolateral (A → B) transport in MDCKII-MDR1 cell monolayers was determined by addition of 0.65 mL of a tritiated compound in concentrations of 0.01, 1 and 50 µM to the apical compartment in triplicate. A volume of 1.8 mL of HBSS/HEPES was added to the basolateral compartment. To determine basolateral to apical (B → A) transport, 1.95 mL of a tritiated compound at concentrations of 0.01, 1 and 50 µM was added to the basolateral compartment and 0.5 ml of HBSS/HEPES to the apical compartment. A 150 µL aliquot of the apical (A → B) or basolateral (B → A) compartment was taken at the start of the experiment to measure the initial concentration and to calculate the recovery of radioactivity. Incubations were performed on a rocker platform (rotation approximately 60 rpm) at 37 °C for 2 h in a humidified incubator containing approximately 95% air/5% CO_2_. Aliquots of the apical and basolateral samples (150 and 1600 µL for A → B and 400 and 150 µL for B → A, respectively) and samples of the initial concentration were mixed with 10 mL of liquid scintillant Ultima GoldTM (PerkinElmer Inc.). Radioactivity was determined by LSC on a Tri Carb 3100TR liquid scintillation counter using QuantaSmart^TM^ software (PerkinElmer Inc.) in which all counts were converted to disintegrations per minute (DPM) using tSIE/AEC (transformed spectral index of external standards coupled to automatic efficiency correction). Background values were measured for each sample sequence using liquid scintillant without test samples. Recovery of radioactivity was calculated as percentage of measured total radioactivity after the experiment versus initial radioactivity.

Subsequently, the effects of MDR1-inhibitors ketoconazole and tariquidar on the bidirectional transport of the tested tritiated compounds were examined. Stock solutions of the inhibitors ketoconazole (10 mM) and tariquidar (10 mM) were prepared in DMSO. On the day of the experiment, inhibitors were added to HBSS/HEPES solutions of the tritiated test compounds in a concentration of 0.01 µM or [^3^H]digoxin in a concentration of 0.05 µM to reach a final concentration of 25 µM ketoconazole or 10 µM tariquidar. These concentrations were based on IC_50_ values that are known to block P-gp completely [16,25]. The assay was performed in an identical manner as described above, except that all apical (in A → B experiment) and basolateral (in B → A experiment) solutions contained 25 µM ketoconazole or 10 µM tariquidar from the start of the experiment, keeping the concentrations of test compounds (0.01 or [^3^H]digoxin 0.05 µM, 10 kBq/mL) and organic solvent concentration (≤1%) constant for all samples.

#### 4.4.4. Calculations

*P*_app_ representing A to B permeability and B to A of a test compound was calculated according to the following equation:

*P*_app_ = (dQ/dt)/(A × C_0_) (1)

where dQ/dt indicates the appearance rate of a test compound at the receiver side calculated from total DPM at *t* = 120 min, corrected for background (DPM/s). A is the surface area of the filter insert (cm^2^) and C_0_ the measured initial concentration, calculated from total DPM on the donor side at *t* = 0 min (DPM/L).

To determine the ER for the test compounds, the following equation was used:

ER = *P*_app_ B → A/*P*_app_ A → B (2)

### 4.5. In Vitro Experiments with Non-Radioactive Molecules

The transport assay with non-radioactive compounds in Caco-2 cells was performed in 96-well plates as described previously [38]. The initial concentration of the test compounds in the acceptor well was 10 µM and the concentration in the receiver well was measured after 2 h using UV spectroscopy. Samples from one compound were pooled in order to have a sufficient volume for the UV detection.

The calcein-AM experiment was performed in MDCKII-MDR1 cells according to methods described previously [39]. Calcein cell accumulation in the absence and in the presence of test compounds (0,1, 1, 10, 30, 50 and 100 µM) was evaluated and the basal level of fluorescence was measured in untreated cells. EC_50_ values were determined by fitting the fluorescence increase (in %) versus log[dose].

### 4.6. Animal Experiments with [^18^F]MC198 and [^18^F]KE64

All animal studies were in compliance with the local ethical guidelines. Protocols (DEC 6456C) were approved by the Institutional Animal Care and Use Committee of the University of Groningen. Male FVB wild type mice (33 ± 2 g, 11–12 weeks) and *Mdr1a/b*^(*−/−*)^*Bcrp1*^(*−/−*)^ constitutive knockout mice (31 ± 3 g, 11–12 weeks) developed from the FVB line were purchased from Taconic (Hudson, NY, USA). After arrival, animals were housed individually and acclimatized for at least 7 days in the Central Animal Facility of the University Medical Center Groningen before experiments. Mice had access to food and water *ad libitum* and were kept under a 12 h light-dark cycle. During experiments, mice were anesthetized with 2% isoflurane in medical air and kept at a constant temperature using electronically controlled heating pads.

#### PET Procedure and Data Analysis

Mice were injected with radiotracers (3.5 ± 1.6 MBq, 0.1 mL, [^18^F]MC198 or [^18^F]KE64) in the penile vein under isoflurane anesthesia. Injections were performed with the mice in the microPET camera (microPET Focus 220, Siemens Medical Solutions, Malvern, PA, USA), simultaneously starting a 30 min dynamic emission scan. After each emission scan, a transmission scan of 515 s with a ^57^Co point source was performed to correct the emission data for attenuation and scatter. Mice were terminated by cervical dislocation. Several organs and tissues were excised, weighed and radioactivity was measured in a γ-counter (LKB Wallac, Turku, Finland). Radioactivity accumulation in the organs was expressed as SUV, using the following formula: [tissue activity concentration (MBq/g)]/[injected dose (MBq)/body weight (g)]. Metabolites in the terminal plasma and brain samples were determined using a radio-TLC method described elsewhere [20]. TLC-plates were eluted with ethylacetate/methanol 9:1 *v*/*v* (R_f_ [^18^F]MC198 = 0.45, R_f_[^18^F]KE64 = 0.62). PET data were reconstructed as described previously [20]. Inveon Research Workplace software version 4.0 (Siemens, Erlangen, Germany) was used for data analysis. All frames were summed, and PET images were co-registered with an MRI template [40]. A whole brain volume of interest (VOI) based on the MRI template was generated. Radioactivity concentrations were converted to SUV values and plotted as time-activity curves (TAC).

### 4.7. Statistical Analysis

The statistical significance of differences between two groups was calculated by two-sided unpaired Student’s *t* test, using IBM SPSS Statistics version 22 (Armonk, NY, USA). One-way analysis of variance (ANOVA) with Bonferroni correction was used to assess differences between three or more groups. A *p* value of less than 0.05 was considered statistically significant.

## 5. Conclusions

In vitro prediction of in vivo results is always challenging, but this is especially the case for P-gp ligands, as their behavior appears to be concentration dependent. Neither the bidirectional transport assay in Caco-2 cells with the non-radioactive compounds verapamil, *N*-FeVer, *O*-FeVer, MC224, MC225, MC198 and KE64 in a single concentration nor the calcein-AM inhibition assay in MDCKII-MDR1 cells was sufficiently correlated with in vivo results in rodents by PET imaging. The predictive value of the in vitro data improved when the bidirectional transport assay in MDCKII-MDR1 cells was performed with tritium-labeled compounds at three different concentrations and in combination with P-gp inhibitors. Based on the data of all experiments, the order of in vitro substrate strength was: *O*-FeVer > verapamil > *N*-FeVer > MC225 ~ MC198 > MC224 ~ KE64 and in vivo: verapamil > *O*-FeVer > MC225 > *N*-FeVer > MC198 > KE64 > MC224.

## Figures and Tables

**Table 1:** In vitro and in vivo results of the P-gp radiotracers tested.

	In Vitro			In Vivo				
	MDCKII-MDR1 Cells							
Compound	ER 0.01 µM	Calcein-AM EC_50_ (µM)	Substrate In Vitro	Brain-to-Plasma Knockout or Treated/Control		AUC Whole Brain Knockout or Treated/Control		Substrate In Vivo
				Mice	Rats	Mice	Rats	
Verapamil	5.5	1.1	+	7.1 ^2^		6.2 ^1^ 3.7 ^2^	7.1 ^3^	+
*N*-FeVer	2.8	1.1	+		5.8 ^3^	1.2 ^1^	3.7 ^3^	+
*O*-FeVer	37	2.4	+	6.5^1^		5.9 ^1^	2.4 ^3^	+
MC224	1.8	0.54	−	1.4^2^		1.1 ^2^		−
MC225	2.4	0.35	+	4.3 ^1^ 3.2 ^2^	1.9 ^4^	1.8 ^1^ 2.0^2^	3.7 ^4^	+
MC198	2.1	0.27	+	2.2 ^2^		1.7^2^		+
KE64	1.9	0.014	−	2.1 ^2^		1.6^2^		+

Definition as substrate (+) in vitro was ER > 2 and in vivo brain-to-plasma or AUC ratio > 2 in at least one strain. ^1^ *Mdr1a/b*^(*−/−*)^ 60 min scan (verapamil, *N*-FeVer and *O*-FeVer) or 30 min scan (MC225, 45 min time point in brain-to-plasma) [19,20]. ^2^ *Mdr1a/b*^(*−/−*)^*Bcrp1*^(*−/−*)^ 30 min scan, 45 min time point in brain-to-plasma [20]. ^3^ Wistar rats treated with tariquidar (15 mg/kg). *N*-Fever, 15 min time point in brain-to-plasma ratio and treatment with tariquidar 15 min prior to the radiotracer. All other values, treatment 30 min before tracer injection and 60 min PET scan [19]. ^4^ Sprague-Dawley rats treated with tariquidar (8 mg/kg) 30 min prior to the radiotracer, 60 min scan [21].
